# ETV2 mediates endothelial transdifferentiation of glioblastoma

**DOI:** 10.1038/s41392-018-0007-8

**Published:** 2018-02-09

**Authors:** Chengjian Zhao, Gustavo A. Gomez, Yuwei Zhao, Yu Yang, Dan Cao, Jing Lu, Hanshuo Yang, Shuo Lin

**Affiliations:** 1 Department of Molecular, Cell and Developmental Biology, University of California - Los Angeles, Los Angeles, CA 90095 USA; 2 Blood Research Laboratory, Chengdu Blood Center, Sichuan, China; 3 State Key Laboratory of Biotherapy and Cancer Center, West China Hospital, Sichuan University, and Collaborative Innovation Center for Biotherapy, Sichuan, China

## Abstract

Glioblastoma multiforme (GBM) is characterized by extensive endothelial hyperplasia. Recent studies suggest that a subpopulation of endothelial cells originates via vasculogenesis by the transdifferentiation of GBM tumor cells into endothelial cells (endo-transdifferentiation). The molecular mechanism underlying this process remains poorly defined. Here, we show that the expression of ETS variant 2 (ETV2), a master regulator of endothelial cell development, is highly correlated with malignancy. Functional studies demonstrate that ETV2 is sufficient and necessary for the transdifferentiation of a subpopulation of CD133+/Nestin+ GBM/neural stem cells to an endothelial lineage. Combinational studies of ChIP-Seq with gain-of-function RNA-Seq data sets suggest that ETV2, in addition to activating vascular genes, represses proneural genes to direct endo-transdifferentiation. Since endo-transdifferentiation by ETV2 is VEGF-A independent, it likely accounts for the observed resistance of GBM tumor cells to anti-angiogenesis therapy. Further characterization of the regulatory networks mediated by ETV2 in endo-transdifferentiation of GBM tumor cells should lead to the identification of more effective therapeutic targets for GBM.

## Tumor cells: a dangerous transformation

A key regulator of endothelial cell development contributes to malignant progression in glioblastoma by promoting a switch in cell fate. Shuo Lin at the University of California Los Angeles, and colleagues, found high levels of ETS Variant 2 (ETV2) in tumor samples from patients with fast-growing, malignant brain cancer. Stimulating Etv2 gene expression in glioblastoma neural stem cells prevented the activation of genes involved in neural differentiation and activated genes required for vascular development. This transformation of tumor cells into endothelial cells contributes to the formation of new blood vessels that supply oxygen and nutrients to the tumor. Understanding how this process is regulated will aid the development of new therapeutic strategies not only against glioblastoma but also other types of cancer, such as breast cancer and leukemia, in which a similar tumor to endothelial cell switch has been described.

## Introduction

Glioblastoma multiforme (GBM) is the most frequent malignant brain tumor in adult humans and one of the most deadly malignancies, with a median patient survival of 12–15 months.^1^ Despite aggressive treatment at diagnosis, which typically consists of resection of the tumor, followed by concurrent and/or subsequent radiation and chemotherapy with temozolomide, the tumor almost invariably recurs and progresses. The hallmark that distinguishes glioblastoma from all other glial tumors is the extensive microvascular proliferation in conjunction with necrosis.^2, 3^ Thus, treatment with antiangiogenic agents holds great promise for this highly vascularized malignant tumor. However, even with the most current therapies targeting angiogenesis, such as the use of inhibitors or antibodies against VEGF-A, patient relapse still occurs, and only a transient shrinkage of the tumor is observed. More importantly, there is no effect on the patient survival rate.^4^ It is believed that the negligible effect of antiangiogenic therapies on GBM is likely due to the unique vascularization process unique to this type of tumor.^5^

Similar to that of other tumors, the GBM vasculature is morphologically and functionally different from normal tissues. It is tortuous, highly disorganized, highly permeable, and intact with endothelial walls, resulting in deficient pericyte coverage and disruption of the blood–brain barrier.^6^ In addition, the GBM vasculature has a unique feature, so-called “glomeruloid tufts,” that exhibit aggressive proliferation of endothelial cells (ECs) compared with those of anaplastic glioma (grade III).^7^ Recently, several research groups have indicated that these aggressive proliferating ECs in GBM are malignant and directly derived from GBM tumor cells.^2, 8, 9^ These tumor-derived ECs (TDECs), ranging from 20 to 90% of the tumor vasculature, are genetically abnormal and refractory to inhibition of both VEGF-A and basic fibroblast growth factor (bFGF and FGF-2) pathways. Further investigation revealed that GBM TDECs are preferentially localized in deep hypoxic areas of GBM, suggesting a possible role of hypoxia in forcing the putative glioma cells toward endothelial-like cells. However, key regulators that convert GBM tumor cells to TDECs remain to be identified.

The activation of certain genes in embryonic stem (ES) cells has been similarly observed in various human epithelial cancers, including breast, liver, gastric, lung, brain, and bladder cancer.^10, 11^ Combined expression of multiple ES-specific genes confers tumor cells an ES-like phenotype. It is therefore hypothesized that activation of specific core transcriptional regulators that are normally active in ES cells, such as SOX2 and OCT4 and their downstream target genes such as NANOG and DPPA2, might afford tumor cells the ability to undergo dedifferentiation and reactivation of a specific developmental program, including vasculogenesis. Supporting this hypothesis is an analysis of large-scale data sets by Weinberg and colleagues highlighting the top 100 genes that are highly correlated between ES and grade IV glioma cells, consisting of genes encoding developmental regulators such as Sox and Ets proteins.^10^

Although several Ets proteins and many Ets DNA-binding sites have been implicated in vascular development,^12–14^ ETV2 is the transcription factor that plays a central role in specifying endothelial cells.^12^ More importantly, we and others have established that overexpression of ETV2 is sufficient to transdifferentiate several somatic cells into functional endothelial cells, including human amniotic cells, fibroblasts, and myoblasts.^15–17^ To determine whether Etv2 also plays a role in the pathogenesis of the unique tumor vasculature of GBM, we analyzed tumor samples from 222 patients by immunohistochemistry and detected a higher level of ETV2 specifically in high-grade glioma (grade III–IV GBM). Co-staining of endothelial markers (CD31 and CD34) with ETV2 revealed preferential expression of ETV2 in GBM tumor vessels and neighboring tumor cells. Functional studies by in vitro and in vivo ETV2 overexpression and gene knockdown experiments indicated that ETV2 alone was sufficient and necessary for endo-transdifferentiation of GBM neural stem cells but not for differentiated GBM cells. RNA-Seq and Chip-Seq results suggest that ETV2 likely inhibit neural differentiation through direct binding to and downregulation of Wnt3a and GSX1, which are important genes required for neural differentiation. In conclusion, these findings establish ETV2 as a key factor involved in the vasculogenesis of GBM and suggest potentially new targets for GBM therapy.

## Results

### Overexpression of ETV2 induces endothelial differentiation from neural cells in zebrafish brain

**Fig. 1:**
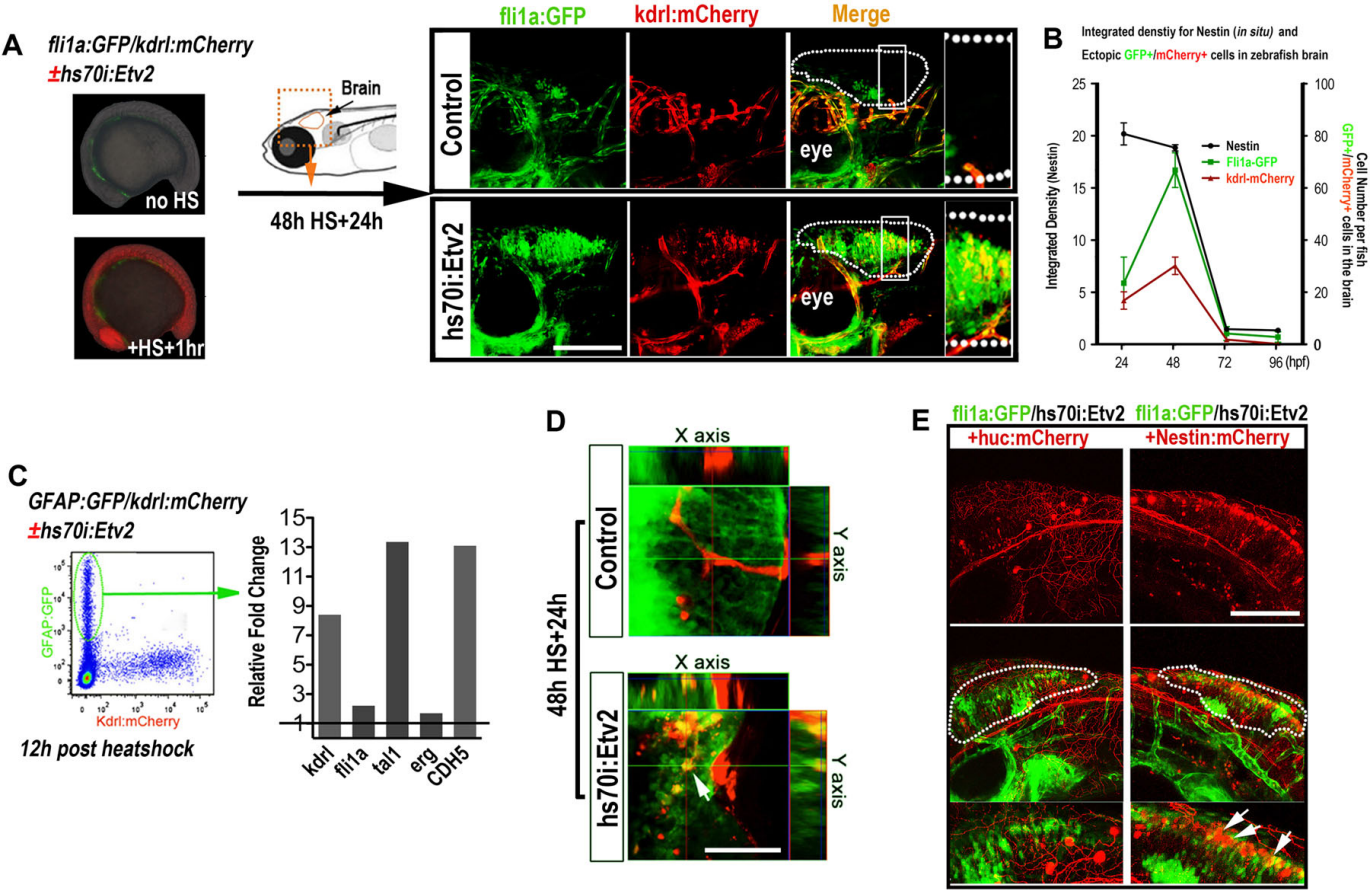
ETV2 induces ectopic endothelial signature in neural stem cells in zebrafish brain. **a** fli1a:GFP/kdrl:mCherry/hsp70i:etv2 embryo at 15 h post fertilization (hpf). Embryos heat shocked at 15 hpf exhibited strong ubiquitous Etv2-mCherry expression at 16 hpf (+HS+1 h). Embryos were then heat shocked at 48 hpf, and by 24 h post heat shock (72 hpf), robust ectopic GFP and mCherry expression (highlighted in dotted white circles) was present in the brain. The yellow dotted box in the zebrafish diagram indicates the imaging area, and areas in white boxes in the merged panels are magnified on the right. Scale bar: 100 μm. **b** The response to ETV2 overexpression is developmentally restricted. fli1a:GFP/kdrl:mCherry/hsp70i:etv2 embryos heat shocked (HS) at 24, 48, 72, and 96 hpf exhibited decreasing numbers of ectopic mCherry+ and GFP+ cells in the brain. Heat shock at 72 and 96 hpf did not induce ectopic mCherry+ or GFP+ expression in the brain. **c** GFAP+ neural stem cells responded to ETV2 expression. GFAP-GFP cells were isolated by FACS from 60 hpf GFAP:GFP/kdrl:mCherry/hsp70i:etv2 embryos (+HS+12 h). qPCR for kdrl, fli1a, tal1, erg, and CHD5 in the isolated GFAP+ cells versus the heat shocked control (GFAP:GFP/kdrl:mCherry). **d** Confocal images showing a kdrl-mCherry+/GFAP-GFP+ neural stem cell (arrow). Scale bar: 50 μm. **e** Ectopic fli1a-GFP+ cells shared the same pattern as the nestin-mCherry+ neural stem cells, and they did not co-localize with huc-mCherry+ mature neural cells. nestin:mCherry or huc:mCherry plasmids were injected into the fli1a:GFP/hsp70i:etv2 embryos at the one-cell stage, and the embryos were then heat shock at 48 hpf and imaged at 72 hpf. Dashed circles highlight ectopic GFP cells; arrows indicate partial nestin-mCherry+/fli1a-GFP+ cells. Scale bar: 100 μm

We previously found that ETV2 overexpression is sufficient to reprogram skeletal muscle cells into functional ECs in zebrafish embryo (~20–24 hpf).^16^ We also sought to test whether ETV2 overexpression could reprogram other somatic cells into the endothelial lineage in vivo. For this purpose, hsp70l:Etv2/kdrl:mCherry/fli1a:GFP triple transgenic zebrafish embryos were heat shock induced to ubiquitously express ETV2 at different time points (24 h, 48 h, 72 h, and 96 hpf) and analyzed for ectopic kdrl-mCherry and fli1a-GFP expression at 24 h post heat shock (hph). This study led to the discovery of robust fli1a-GFP and kdrl-mCherry ectopic expression in the brain of zebrafish that were heat shocked at 24–48 hpf (Fig. 1a). This potential for endothelial induction in the brain by ETV2 expression decreased sharply in embryos at a later developmental stage (at 72, 96 hpf) (Fig. 1b). Gene expression analysis revealed that the endothelial induction time-window overlapped with the expression of nestin, a neural stem cell (NSC) marker, suggesting that NSCs might assume an endothelial fate in the brain. This observation was confirmed by using hsp70l:Etv2/kdrl:mCherry/GFAP:GFP triple transgenic fish (Fig. 1c). GFAP and Nestin expression at this stage has been previously shown to reflect the unique brain NSCs in zebrafish embryo.^18^ At 12 hph, we isolated GFP-positive cells from hsp70l:Etv2/kdrl:mCherry/GFAP:GFP triple transgenic fish by FACS and examined the expression of five endothelial genes (kdrl, fli1a, tal1, erg, and VE-cad). All genes analyzed were significantly induced in the embryos following ETV2 overexpression (Fig. 1c). In addition, at 24 hph, confocal images showed the co-localization of GFAP-GFP^+^/kdrl-mCherry^+^ cells in the brain (Fig. 1d, arrows). These results suggested that NSCs respond to ETV2-mediated endothelial transdifferentiation. To further confirm this finding, we injected Nestin:mCherry and Huc:mCherry plasmid into the hsp70l:Etv2/fli1a:GFP double transgenic zebrafish at the one-cell stage to label the differentiated neurons (Huc-mCherry^+^) and neural stem and progenitor cells (Nestin-mCherry). Zebrafish embryos were then heat shocked at 48 hpf, followed by confocal microscopy analysis. This study revealed that ectopic expression of fli1a:GFP in the brain was frequently overlapped with the nestin:mCherry but not with Huc:mCherry (Fig. 1e). Under the same conditions, ectopic fli1a:GFP expression in the brain was never detected in control embryos (without hsp70I:Etv2). Overall, we confirmed that neural stem and progenitor cells, but not differentiated neural cells, were able to respond to ETV2 induction and transdifferentiate into endothelial cells.

### ETV2 is highly expressed in high-grade human glioma

**Fig. 2:**
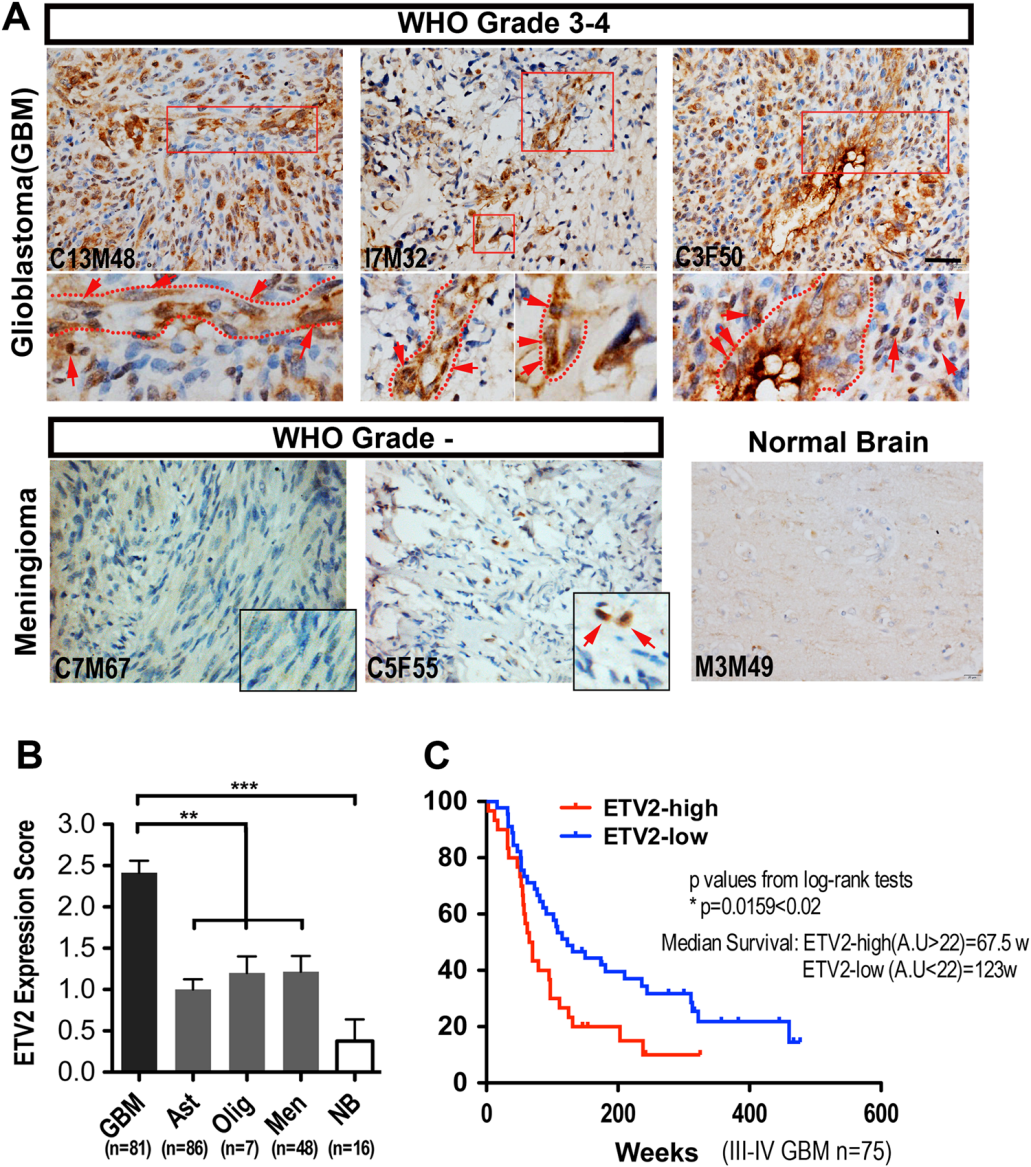
ETV2 expression is preferentially detected in high-grade human gliomas. **a** IHC staining for ETV2 of human brain tumors and normal brain tissues (GBM grade III–IV, *n* = 81; astrocytoma grade I–II, *n* = 86; meningioma grade I–II, *n* = 48; oligodendroglioma grade I–II, *n* = 7 and normal brain tissues, *n* = 5); areas in dashed boxes are magnified below; ETV2+ cells were highlighted by dashed circles or arrows. See also Fig. S1. Scale bar: 50 μm. **b** Quantitative analysis of the expression level of ETV2 in different human brain tumors and normal tissues. **c** Kaplan–Meier analysis of patient OS according to the ETV2 expression level in high-grade brain tumors (*n* = 77). From the TCGA database, a total of 77 high-grade brain tumor patients were divided into ETV2-high (*n* = 30) or ETV2-low (*n* = 47) groups, in which the cutoff value was the average ETV2 expression level of all patients

Based on the observation that ETV2 can induce vascular gene expression in NSCs in the zebrafish brain and that ETV2 is able to transdifferentiate several somatic cells into a functional endothelial lineage,^15–17^ we rationalized that it could also play a role in GBM vasculogenesis. As an initial step to test this hypothesis, we first investigated the expression of ETV2 in clinical brain tumor specimens, including glioblastoma (GBM grade III–IV, *n* = 81), astrocytoma (grade I–II, *n* = 86), meningioma (grade I–II, *n* = 48), and oligodendroglioma (grade I–II, *n* = 7). The demographic information is summarized in the Supplementary Information (Tables S1 and S2). The expression levels of ETV2 in different brain tumor and normal brain (*n* = 16) samples were analyzed by IHC (immunohistochemistry) and western blotting. As shown in Fig. 2a, ETV2 was highly expressed in high-grade glioma (grade III–IV GBM). Occasionally, ETV2 expression was detected in low-grade brain tumors (Fig. 2a and Fig. S1). The intensity of ETV2 expression was scored using a four-step scale: no staining, 0; weak staining, 1+; moderate staining, 2+; and strong staining, 3+. Scores of 0 and 1+ were classified as negative, and scores of 2+ and 3+ as positive. Among the 222 tumor samples, positive ETV2 expression was observed in 76.5% (62/81) of GBM and in 39.7% (56/141) of the low-grade brain tumor (astrocytoma, oligodendroglioma, and meningioma) but not in normal brain tissues (*P* < 0.0001, Fig. 2b). Intriguingly, we observed that some ETV2+ GBM tumor cells appeared to have vascular structures (Fig. 2a, dotted lines), while ETV2+ tumor cells in low-grade brain tumors were randomly distributed (Figs. 2a and S1). The expression of ETV2 in high-grade glioma was also confirmed in freshly dissected clinical samples (GBM grade III–IV, *n* = 9; meningioma, *n* = 1; skeletal muscle, *n* = 1) by reverse transcriptase PCR (RT-PCR) and western blotting, as shown in Fig. S1. Together, these data indicate that ETV2 is significantly enriched in high-grade glioma.

We noticed that the ETV2 expression level varied even in high-grade glioma. Therefore, to determine the prognostic value of ETV2, the previously published microarray data and clinical follow-up information from 77 grade III–IV brain tumor samples from M.D. Anderson Cancer Center (MDA) and UCSF were re-analyzed by Kaplan–Meier analysis and the log-rank test.^19^ The demographic information is summarized in Supplementary Information (Table S3). As shown in Fig. 2c, GBM (*n* = 30) with higher ETV2 expression had a median survival of 67.5 weeks, whereas the group with relatively lower ETV2 expression (*n* = 45) had a median survival of 123 weeks (*P* < 0.02). This result indicated a possible prognostic value of the ETV2 level in patients with high-grade glioma. Furthermore, these observations suggest a possible role of ETV2 in GBM malignant progression.

### ETV2-positive GBM tumor cells co-express endothelial genes

**Fig. 3:**
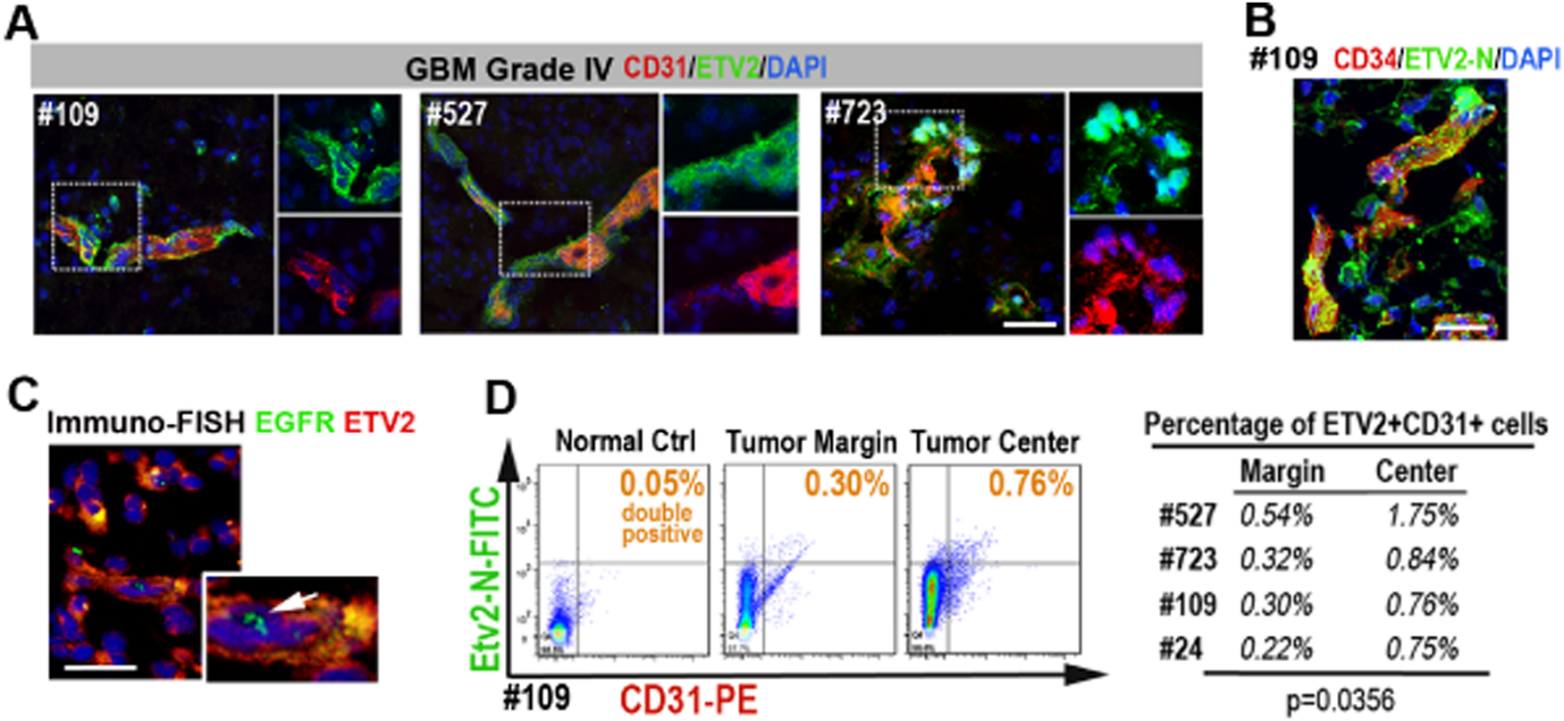
ETV2+ GBM tumor cells display endothelial signatures. **a** Multicolor IF staining for ETV2 and CD31 using three freshly collected grade IV GBM tissue (#109, #527, #723); areas in dotted boxes are magnified on the right. See also Fig. S4. **b** Multicolor IF staining for ETV2 and CD34 using GBM #109; the area in the dotted box is magnified on the right. **c** Immuno-FISH staining indicated that the ETV2+ cells were actually malignant. Scale bars in **a**, **b**, **c**: 50 μm. **d** One representative patient sample for comparison of the percentage of CD31+ ETV2+ cells in the margin areas and deeper areas (3–5 cm from the margin) of the resected GBM tissues; the percentage of ETV2+ CD31+ cells among CD31+ cells is marked **c** and summarized in a table (**c**, right panel)

To determine whether ETV2 expression in GBM indeed had endothelial properties, we analyzed its association with established endothelial markers. We dissected and sectioned four freshly collected grade IV GBM (GBM #109, GBM #723, GBM #527, GBM #24 from West China Hospital). Multicolor immunofluorescence staining (IF staining) showed that many ETV2+ tumor cells were preferentially arranged as vascular-like clusters, notably co-expressing CD31 and CD34 (Figs. 3a, b and S4). IF staining was also performed using low-grade brain tumors and normal human tissues (meningioma, *n* = 2; liver, *n* = 3; intestine, *n* = 2; and muscle, *n* = 3). In low-grade tumor samples, ETV2 expression and co-localization of ETV2 with CD31 were rarely detected (Fig. S2). To validate that ETV2+ EC-like cells were derived from malignant GBM tumor cells, multicolor FISH immunostaining was then performed, and the results showed the multicopies of *EGFR* genes in these cells (Fig. 3c). Hence, these data together indicated a correlation between the expression of ETV2 and CD31+/CD34+ EC-like cells in the GBM tumor.

Flow cytometry analysis of the clinical GBM samples was further performed to assess the frequency of ETV2+ CD31+ cells located in either the tumor margin or deeper regions (3–5 cm from the margin) of the GBM samples. Significantly more ETV2+ CD31+ cells were detected in the deeper regions than in the marginal area (Fig. 3d), consistent with the previous observation that GBM TDECs were preferentially located in deeper hypoxic areas.^9^ We noted that ETV2 expression in the GBM tumor cells was not strictly restricted to the nucleus (Fig. S3). To assure that our finding was genuine, we further evaluated additional ETV2 antibodies that were raised against different regions of human ETV2 protein (ETV2^C-terminal^; ETV2^N-terminal^; ETV2^internal^, detailed information material and methods). All three ETV2 antibodies were found to have similar expression patterns in the clinical GBM tissues (Fig. S4).

### Overexpression of ETV2 reprograms GBM tumor cells into endothelial cells both in vitro and in vivo

**Fig. 4:**
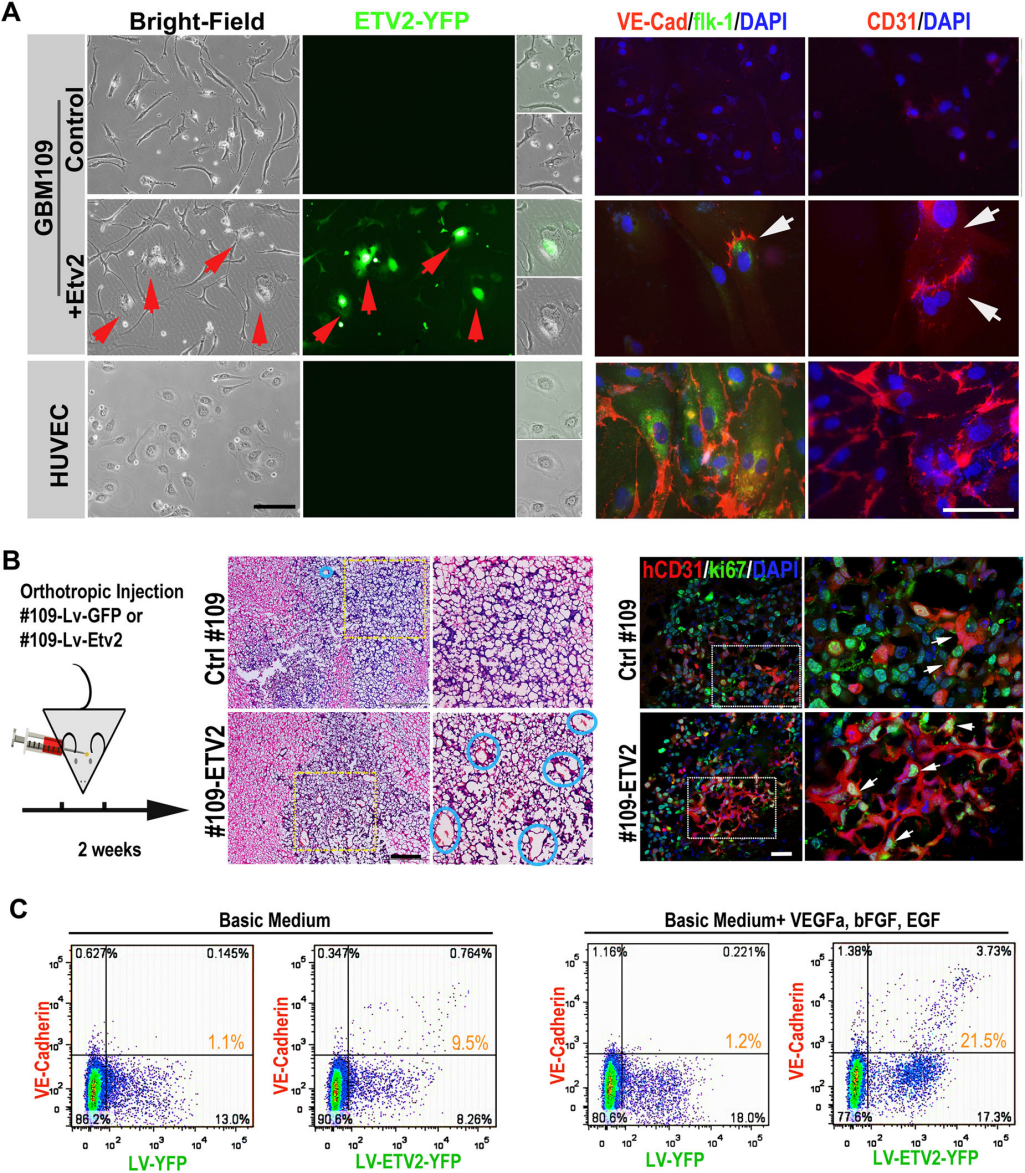
Overexpression of ETV2 reprograms p-GBM tumor cells into endothelial cells in vitro and in vivo. **a** p-GBM cells (#109) were reprogramed into endothelial-like cells 1 week following ETV2 overexpression in vitro. Patient GBM cells were propagated as neurospheres under optimal conditions for neural stem cells. One week after ETV2 overexpression, partial p-GBM cells showed an endothelial morphology (red arrows) and the ability to express endothelial genes (VE-cad, flk-1, CD31, CD34, and tie2, among others) (white arrows). HUVECs were set as the positive control. Scale bar: 50 μm. **b** p-GBM cells (#109) were reprogramed into endothelial-like cells and arranged as vascular tubes in vivo. GBM #109-ETV2 or control GBM #109-YFP cells were orthotopically implanted into the brain of NOD/Scid mice (1 × 10^6^ cells in each mouse, *n* = 5 per group). HE staining indicated the GBM tumors, areas in dotted boxes, are magnified on the right; blue circles indicate tumor blood vessels. **b** IF staining showed the tumor cell-derived ECs (hCD31+/ki67+ arrows). Scale bar: 50 μm. **c** Angiogenic growth factors were dispensable for endo-transdifferentiation of GBM tumor cells, but they were beneficial for TDEC enrichment. GBM #109 tumor cells infected with LV-ETV2-YFP or LV-YFP were cultured in basic endothelial medium free of growth factors or supplemented with VEGFa (50 ng/ml), bFGF (20 ng/ml), and EGF (10 ng/ml) for 5 days. The percentage of VE-Cad+ cells among the successfully infected YFP+ cells is indicated

To test the function of Etv2 in GBM, we constructed a lentiviral vector expressing the ETV2-YFP fusion protein under the control of the EF1α promoter to overexpress Etv2 in GBM tumor cells. To better represent the biological status in vivo, GBM cells from patients and GBM cell lines (GBM #109, GBM #723, GBM #527, GBM #24, A172, U251, U87) were propagated as neural spheres under optimal conditions for neural stem cells.^20^ Empty control lentivirus infection was used as a control. After induction, the cells were cultured in endothelial growth medium (complete EGM) before evaluation. At 1–4 weeks after ETV2 overexpression, RT-PCR and qPCR analysis revealed that endothelial genes were robustly induced in primary GBM cells (#109, #723, #527, and #24) and U87 (Fig. S5). At 10 days post ETV2 overexpression, multicolor immunostaining also showed that ETV2 overexpression in primary GBM tumor cells induced significant expression of endothelial marker genes (kdrl, VE-Cad, and CD31). Additionally, an endothelial-like morphology was observed in these Etv2-overexpressing tumor cells (Fig. 4a).

Next, we tested the endo-transdifferentiation of GBM tumor cells by ETV2 in vivo. GBM #109-ETV2 (ETV2-overexpressing patient GBM cells, 1 × 10^6^, 21% were infected with Lentivirus-ETV2) or GBM #109 (1 × 10^6^, 19% were infected with control Lentivirus) cells were orthotopically implanted into the brain of NOD/Scid mice (*n* = 5 in each group) as previously described.^21^ Two weeks later, the mice were killed, and the brains were sectioned and analyzed by HE as well as multicolor IF. Low magnification of HE staining indicated that the injected tumor cells grew robustly in the brain parenchyma in both groups; however, higher magnification of the sections showed a much higher blood vessel density in the GBM-ETV2 group compared with the control group (Fig. 4b). To confirm that ECs in GBM xenografts were indeed derived from GBM, antibodies specific to human CD31 (hCD31) and ki67 were used for IF analysis (Fig. S6). The results showed significantly more hCD31+ ki67+ tumor cells in the GBM-ETV2 group (14% vs 6%) (Fig. 4b). Additionally, some of the hCD31+ ki67+ tumor cells were arranged as a vascular vessel, although they were more tortuous and abnormal than the normal vasculature (Fig. 4b, arrows). Thus, our in vitro and in vivo studies together established ETV2 as a functional mediator of the endo-transdifferentiation of GBM tumor cells.

It should be noted that ETV2-mediated in vitro endo-transdifferentiation of GBM tumor cells consistently occurred without the addition of endothelial growth factors in the basic EGM growth medium. However, the addition of growth factors (VEGF-A, bFGF, and EGF) to the medium enhanced the transdifferentiation process by approximately twofold (21.5% vs 9.5% TDECs, Fig. 4c). This finding is consistent with a previous report demonstrating that TDEC formation is independent of VEGF-A, bFGF, and EGF.^9^ However, endothelial growth factors are beneficial for the enrichment of TDECs.

### ETV2 is necessary for induction of TDECs in GBM in vivo

**Fig. 5:**
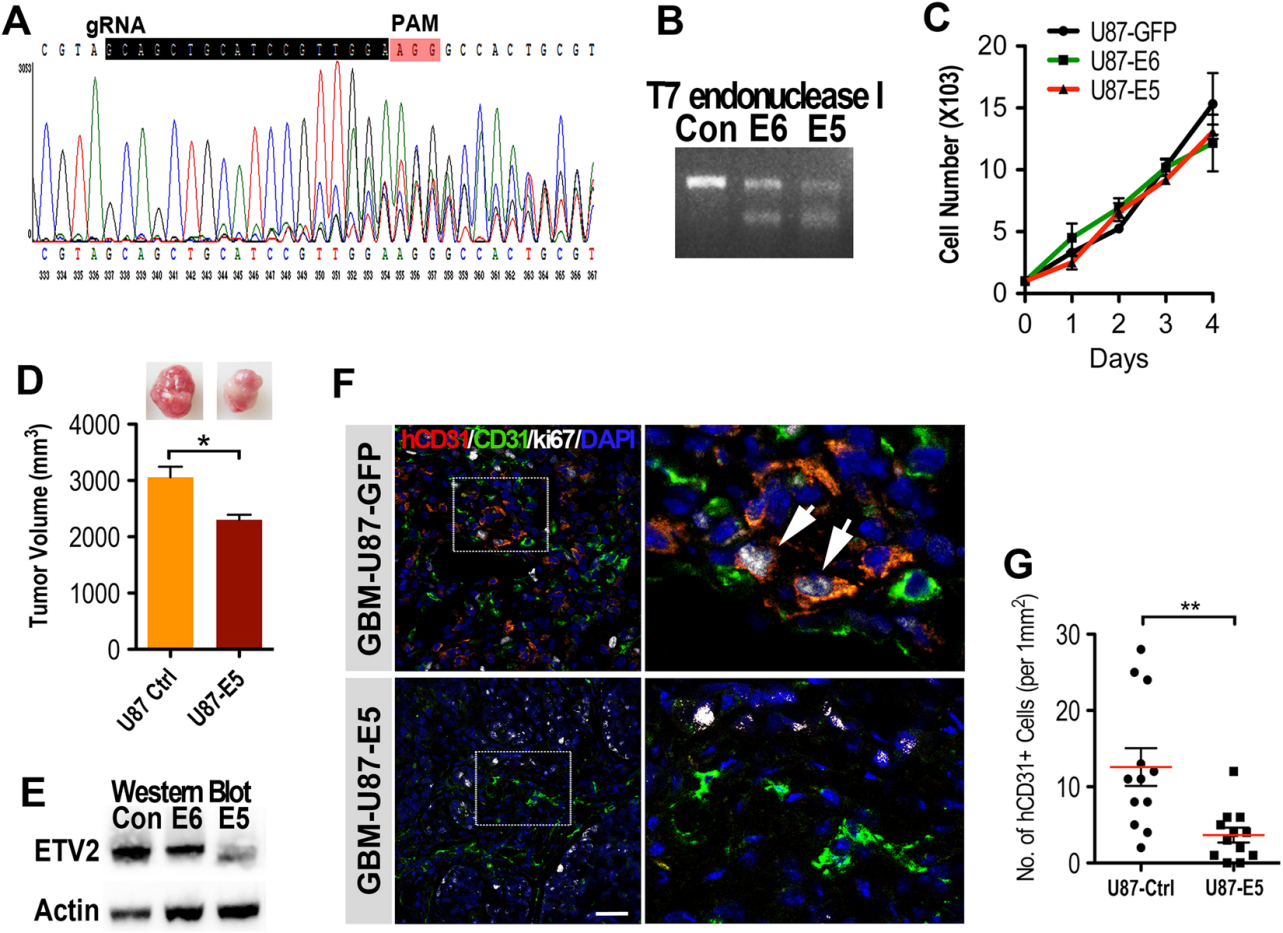
Etv2 knockdown abrogates the ability of endo-transdifferentiation of GBM tumor cells. **a** Etv2 was knocked down using the CRIPR/Cas9 system in GBM-U87 cells. The sgRNA coding sequence is indicated in black, and the protospacer-adjacent motif (PAM) sequence is labeled in red. **b** Two ETV2 knockdown clones (E6 and E5) were selected, and the ETV2-mutation was confirmed by the T7 endo I assay. **c** The growth rate of the GBM tumor cells in vitro was not affected after ETV2 knockdown. **d** The tumor size of GBM-U87-E5 was significantly smaller than that of control GBM-U87 at 2 months after cell implantation (*n* = 5 in each group). **e** The expression level of ETV2 in subcutaneous GBM tumors was analyzed by WB. **f** ETV2 knockdown inhibited the induction of TDECs in GBM tumor cells in vivo. GBM-U87-E5 or GBM-U87 cells were subcutaneously implanted in NOD/Scid mice for 2 months (2 × 10^6^ cells for each mouse, *n* = 5 per group). Multicolor IF staining for ki67, hCD31, and CD31 (recognizing both mouse and human antigen) revealed ki67+/hCD31+/CD31+ TDECs (arrows) in control tumors, but not in U87-E5 tumors. Areas in dotted boxes are magnified on the right. Scale bar: 50 μm

To assess whether ETV2 was required for the induction of TDECs in GBM, the *Etv2* gene was targeted in GBM-U87 cells using the CRISPR/Cas9 system (Fig. 5a). Knockdown of Etv2 in two clones (E5 and E6) was confirmed by the T7E1 assay and sequencing followed by western blotting to demonstrate gene depletion (Fig. 5b, e). These cells appeared morphologically normal and did not show any growth changes in culture compared to normal GBM-U87 cells (Fig. 5c). In addition, we also analyzed the potential off-target effect by the T7 EI assay (Fig. S8). We then subcutaneously engrafted GBM-U87-E5 cells into NOD/Scid mice (5 × 10^6^ cells each), and tumor cells infected with empty CRISPR/Cas9 Lentivirus was used as a control (*n* = 5 in each group). At 2 months after cell injection, the mice were killed, and the GBM tumor mass was resected and analyzed. The tumor size of U87-E5 was significantly smaller than that of the control group, although their growth rates in the dish were similar (Fig. 5c, d). To determine whether the tumor vasculature in U87-E5 tumors was affected, triple IF staining for human CD31 (hCD31), CD31 (detecting both human and mouse antigen), and ki67 was performed. Confocal images indicated that hCD31+/CD31+/ki67+ proliferating TDECs were constitutively induced in the control U87 tumor mass, while no hCD31+/ki67+ TDECs were observed in the tumors in the U87-E5 group (Fig. 5f, g). However, angiogenesis (hCD13-CD31+ ECs penetration) was observed in both groups, suggesting that the loss of ETV2 could block the generation of TDECs originating from the vasculogenesis of GBM but had little or no effect on tumor angiogenesis.

### GBM neural stem-like cells respond to ETV2-mediated endo-transdifferentiation

**Fig. 6:**
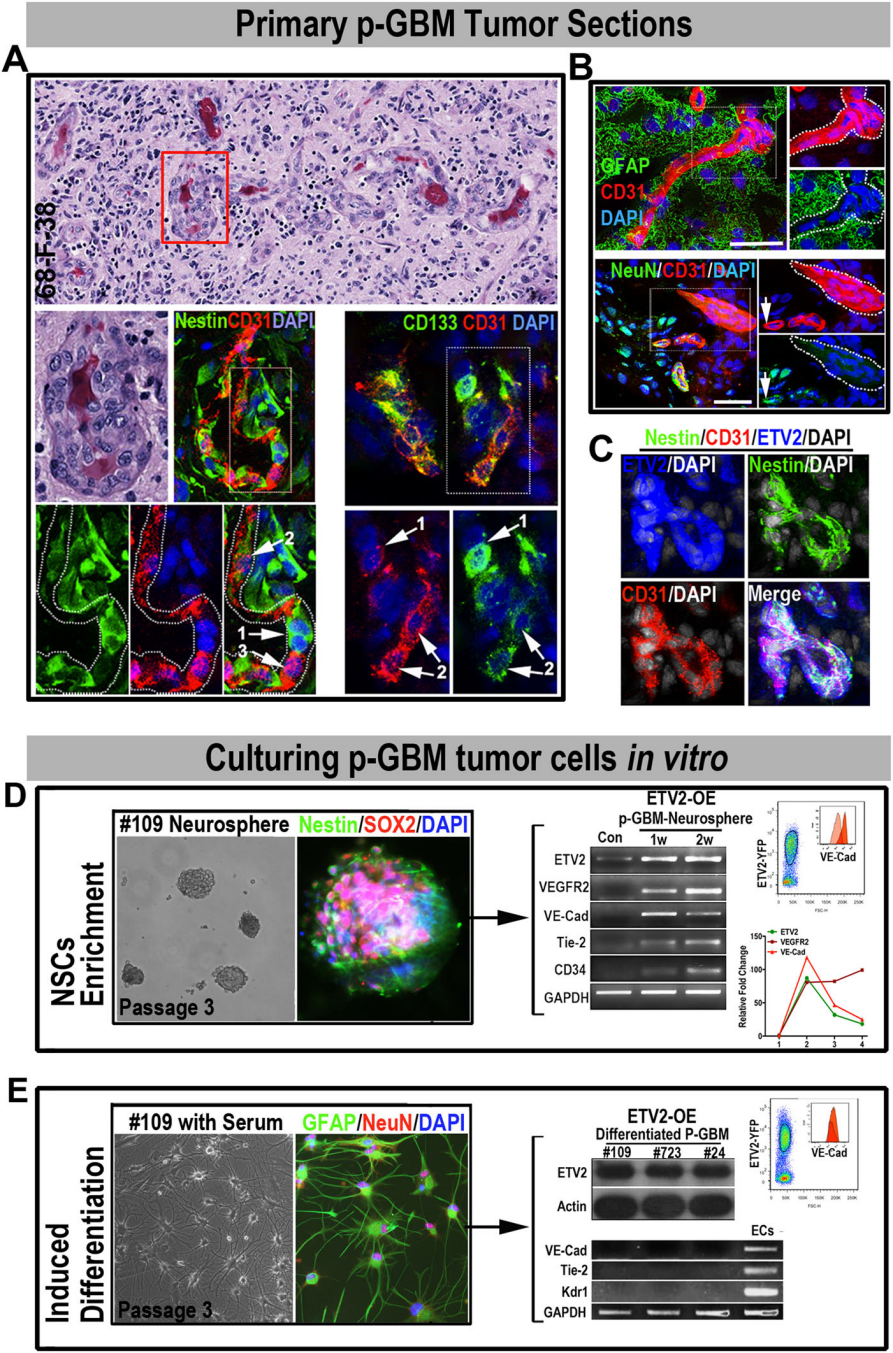
GBM neural stem-like cells are the responding cells for endo-transdifferentiation by ETV2 expression in GBM patients. **a**–**c** A fraction of GBM neural stem cells (nestin+/CD133+) co-expressed with CD31. **a** HE staining of a p-GBM paraffin section showing the GBM blood vessels (red box, **a**). Areas in dotted boxes are magnified below; CD31+ neural stem cells were detected by IF staining: CD133^high^/nestin^high^ CD31^low^ Cells (1, arrows), CD133+/nestin+ CD31+ cells (2, arrows), CD133^low^/nestin^low^ CD31^high^ Cells (3, arrows). **b** Coexpression of CD31 with mature neural maker genes (GFAP and NeuN) was rarely observed. Dashed circles highlight CD31+ cell clusters, arrows indicate NeuN+ CD31+ cells. **c** The nestin+ CD31+ ETV2+ triple-positive GBM cells were consistently detected. **d** ETV2 induced an endothelial signature in GBM neural stem-like cells. GBM neural stem-like cells (sox2+ nestin+, passage 3) were propagated as neurospheres under optimal conditions for neural stem cells. Flow cytometry, RT-PCR, and qPCR tests after 1–3 weeks of ETV2 overexpression indicated the successful induction of endothelial genes in GBM #109 neural stem-like cells. **e** Differentiated GBM tumor cells did not respond to ETV2 overexpression, and differentiated p-GBM tumor cells (GFAP+ NeuN+, passage 3) cultured with serum; RT-PCR and WB assays indicated that the differentiated GBM tumor cells (#109, #723, #24, at 2 weeks post LV-ETV2 infection) were unresponsive to ETV2 overexpression; HUVECs were set as the positive control. Scale bars in **a**, **b**, **c**, **d**, and **e**: 50 μm

GBM is composed of both plastic neural stem-like cells and lineage-committed mature tumor cells. To determine the origin of TDECs in primary patient GBM (p-GBM), we first co-stained for ETV2 with neural stem-like cell markers (Nestin, Vimentin, and CD133) or mature neural tumor cell makers (NeuN and GFAP) in cryosections of p-GBM autopsies. The confocal images showed that fractions of ETV2+ cells were preferentially co-expressed with Nestin, Vimentin, and CD133, while NeuN+/ETV2+ and GFAP+/ETV2+ cells were not detected (Fig. S7, #109). Next, to determine whether GBM neural stem-like cells were the responding cells to endo-transdifferentation in vivo, HE and multicolor IF staining for Nestin, CD133, NeuN, and GFAP with CD31 were performed using primary GBM autopsies. We found that fractions of CD31+ cells were co-stained with the neural stem cell markers nestin and CD133, while GFAP+ CD31+ or NeuN+ CD31+ cells were rarely detected (Fig. 6a, b). Interestingly, we found that among the Nestin+ CD31+ or CD133+ CD31+ cells, high expression of CD31 was mostly associated with low expression of neural stem cell markers (Nestin or CD133) and vice versa (Fig. 6a, arrows), suggesting a certain loss and gain of specific marker genes during the process of endothelial lineage commitment from GBM neural stem-like cells. To correlate the ETV2 expression with the endo-transdifferentiation of GBM neural stem-like cells, we further performed triple immunostaining for ETV2, CD31 with nestin using the cryosections of GBM autopsy samples (Fig. 6c). As expected, triple-positive cells (ETV2+/CD31+/nestin+) were frequently detected, suggesting a correlation between ETV2 expression and the endo-transdifferentiation of GBM neural stem-like cells.

To determine whether the endo-transdifferentiation potential was limited to the plastic GBM neural stem-like cells. GBM neural stem-like cells were enriched in vitro using the neurosphere culture system. Additionally, differentiated GBM tumor cells were propagated in medium from the same GBM patient (#109). The identities of both primarily cultured p-GBM tumor cells were confirmed by analyzing the expression of specific markers (sox2, nestin for GBM neural stem cells; GFAP, NeuN for differentiated GBM tumor cells) (Fig. 6d, e). The GBM tumor cells (after 3 passages) were then infected with LV-ETV2 or control virus and cultured in complete EGM for weeks. The induction of endothelial genes (kdrl, tie2, VE-cad, and CD34) in the GBM tumor cells in both groups was then analyzed using RT-PCR or flow cytometry (Fig. 6d, e). As expected, stable transcription of endothelial genes was only detected in p-GBM cells propagated using the neurosphere culture system, and no endothelial gene transcription activity was detected in serum-cultured p-GBM cells. Together, these findings indicated that the GBM neural stem-like cells were the cells that specifically responded to ETV2 overexpression and had the ability to transdifferentiate into endothelial-like cells.

### ETV2 induces vascular transdifferentiation by directly repressing neural commitment genes

**Fig. 7:**
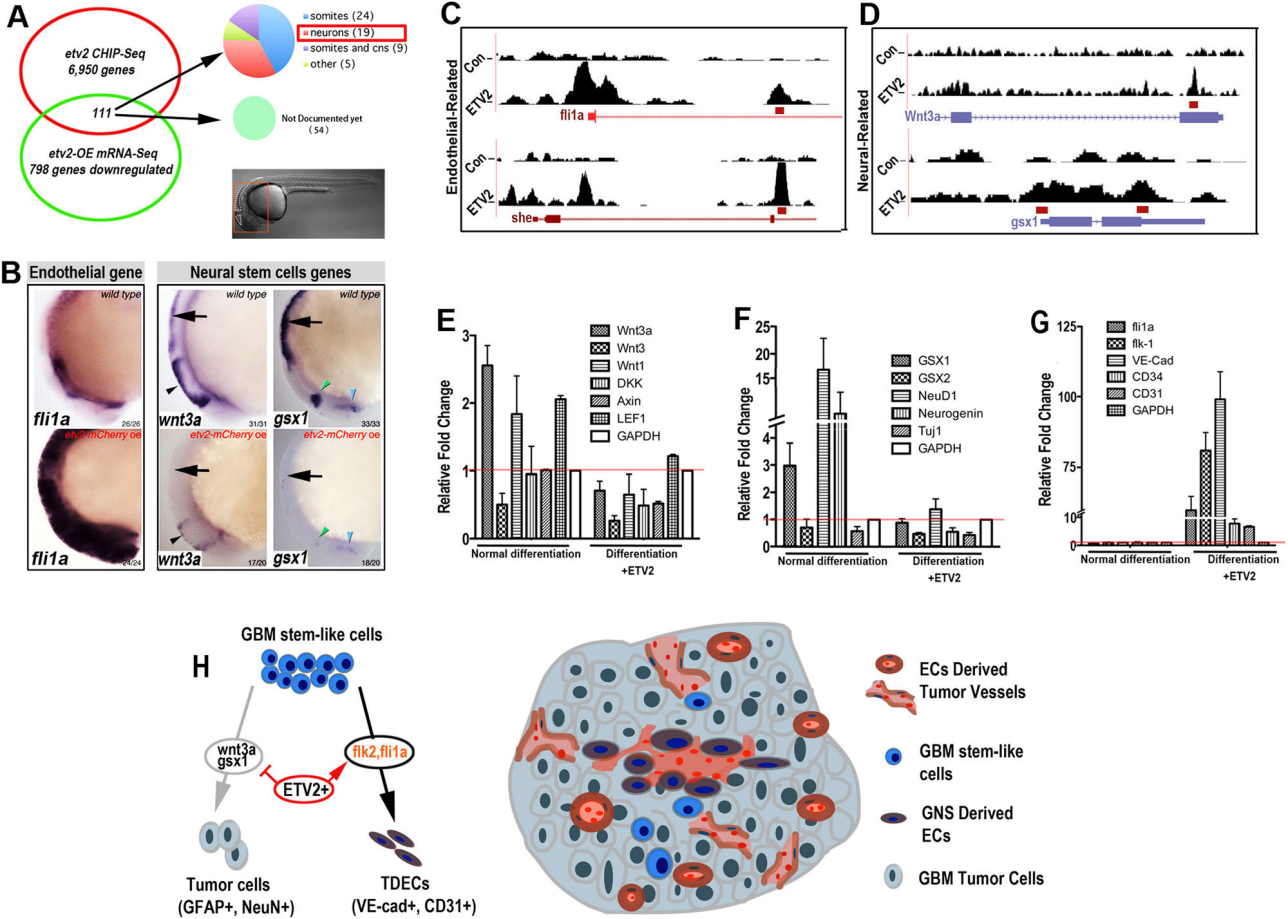
Overexpression of ETV2 inhibits the expression of wnt3a and gsx1. **a**The combination of Chip-Seq and RNA-Seq suggested that 19 neural-specific genes were directly downregulated by ETV2, and 111 of 798 genes were reduced below a twofold cutoff in the RNA-Seq profile at 6 h after heat shock in association with Etv2 ChIP-Seq peaks. The expression of 57 of the 111 genes is available in PubMed databases, and these are broken down as indicated in the pie chart. **b** WISH under different conditions and stage-matched embryos profiled by RNA-Seq for fli1a, wnt3a, and gsx1. The endothelial gene fli1a was ectopically induced in the brain, while expression of the neural genes wnt3a and gsx1 in the hindbrain (arrows) and midbrain (arrowheads) was greatly inhibited. **c**, **d** ChIP-Seq binding profiles of control (top track) and etv2-OE (bottom track) at the fli1a, she, wnt3a, and gsx1 locus. **e**–**g** Serum-induced differentiation of GBM #109 neural stem-like cells with or without ETV2-OE. Evaluation of Wnt activity-related genes (**e**), neural genes (**f**), and endothelial genes (**g**) at 72 h post-virus infection (LV-ETV2 or control lentivirus). **h** Schematic diagram showing the mechanism of ETV2-involved reprogramming of GBM stem-like cells into endothelial-like cells. ECs endothelial cells, TDECs tumor-derived ECs

Reprograming GBM neural stem-like cells into the endothelial lineage is likely a result of both the activation of endothelial differentiation genes and inhibition of neural specification genes. To understand this process, we performed a comparative analysis of ChIP-Seq data sets with gain-of-function RNA-Seq data sets generated in vivo using zebrafish embryos. We examined genes that were repressed by Etv2 overexpression and identified 111 genes with ChIP-Seq peaks, among which a total of 57 genes have been documented and 20 are specifically expressed in the nervous system (Fig. 7a and S9, Supplementary bioinformatics material and method). Among the 20 putative neurogenic-related targets of ETV2, *wnt3a* and *gsx1* have been previously shown to be key genes enhancing GBM/neural stem cell differentiation and neuronal maturation (Fig. 7c, d).^22, 23^

To examine the expression of *wnt3a* and *gsx1* in the brain microenvironment following ETV2 induction, whole-mount in situ hybridization (WISH) was performed 16 h post Etv2 overexpression. We also tested the vascular gene fli1a, for which the RNA-Seq profiling showed induction by ETV2 and ChIP-Seq showed a binding peak in the ETV2 group. We found, compared with the wild-type control, that the expression of both *wnt3a* and *gsx1* were nearly abolished in the brain. Concomitant with this repression, the normally vascular-restricted expression of *fli1a* was ectopically induced throughout the brain following ETV2 overexpression (Fig. 7b). To further extend our findings, we measured the mRNA levels of *gsk1* and *wnt3a* as well as several related neurogenic genes *NeuD1*, *Neurogenin*, and *Tuj1*, along with the endothelial genes *kdrl*, *fli1a*, *VE-cad*, and *tie2* in GBM neural stem cells at 48 h after serum-induced differentiation with or without ETV2 overexpression. As expected, exposure to serum induced the neurogenic differentiation of GBM neural stem cells with enhanced expression of *gsk1* and *NeuD1* and Neurogenin, but not *gsk2*, which is consistent with a previous study showing that *gsk1* promotes NSC maturation and the acquisition of neuronal phenotypes, partially through the downregulation of *gsx2*.^23^ However, ETV2 expression dramatically inhibited the expression of *gsk1* and neural genes *NeuD1* and *Neurogenin*, while it upregulated the expression of *kdrl* and *VE-Cad* (Fig. 7f, g), suggesting a possible mechanism for the endo-transdifferentiation of GBM via the repression of neurogenic specification. Similarly, since the Wnt pathway is also related to the neural differentiation of GBM neural stem cells, we examined the expression of the related genes *wnt3a, wnt3*, *wnt1*, *DKK1*, *Axin*, and *LEF1* during serum-induced differentiation with and without ETV2 overexpression. Similarly, we found that serum exposure induced the expression of *wnt3a*, *wnt1*, and *LEF1*, confirming the activation of the wnt pathway during neural lineage specification. With ETV2 overexpression, as expected, we found that *wnt3a* expression was significantly inhibited (Fig. 7e). Overall, these results suggest that direct binding and inhibition of *Gsx1* and *wnt3a*, as well as the activation of vascular-specific positive regulators, contribute to ETV2-mediated endo-transdifferentiation of GBM neural stem cells (Fig. 7 model).

## Discussion

Tumor vascularization is important for tumor initiation, dormancy, progression, and metastasis. For many years, tumor vascularization has been explained merely by the ingrowth of new vessels into the tumor from preexisting vessels through angiogenesis; however, in recent years, additional mechanisms have been recognized. The possibility of EC differentiation directly from malignant tumor cells has been proposed in lymphoma, myeloma, chronic myeloid leukemia (CML), breast cancer, neuroblastoma, and glioblastoma.^24–26^ These alternative mechanisms to generate tumor vessels perhaps explain the largely ineffective outcome of clinical trials in which antiangiogenic agents (generally targeting VEGF-A) are used in conjunction with chemotherapy. These trials only showed a transient increase in patient survival with advanced solid tumors, with most patients ultimately succumbing to tumor progression. According to one recent study, tumor cell-derived ECs were refractory to inhibition of both the VEGF-A and basic fibroblast growth factor (bFGF and FGF-2) pathways.^9^ In fact, it is well established that tumor endothelial cells participate in the recruitment of stromal cells, including myeloid cells and bone marrow-derived cells, to generate a VEGF-A-independent pathway of tumor resistance to antiangiogenic treatment.^27^ Angiogenesis inhibitors block outside tumor vessels from growing into the solid tumor, thus terminating the supply of nutrients feeding tumor growth. However, there is a growing concept that endothelial cells are not merely passive conduits, but have an instructive role, releasing specific growth factors and inflammatory and protumorigenic cytokines, leading to the mobilization of other cells that can promote tumor growth and affect the response to therapy.^28^ It is possible that the transdifferentiation of tumor cells into endothelial cells can produce these signaling centers to favor tumor growth and resistance to antiangiogenic therapies. To develop more effective therapeutics, these tumor-derived ECs must also be targeted. Here, we identified Etv2 as a key mediator regulating the transdifferentiation of GBM cells into endothelial cells. Not only does this reveal a molecular mechanism for the production of treatment-resistant endothelial cells in GBM patients, but it also provides a potential therapeutic target. It is highly desirable to identify agents or approaches that block the function of Etv2 or proteins involved in its function.

In normal ES cell differentiation, ETV2 acts downstream of BMP, Notch and Wnt signaling for endothelial lineage specification. Inhibition of BMP, Notch, and Wnt signaling pathways greatly decreases the generation of kdrl+ cells and expression of ETV2, while overexpression of Etv2 in ES cells rescues the generation of kdrl+ cells.^29^ All these signaling pathways are active and critically implicated in tumor stem cell fate determination during the pathological progression of GBM.^30, 31^ As a transcription factor, ETV can be difficult to target. First, all the transcription factors are positively charged, requiring the inhibitor candidates to be negatively charged. However, charged pharmaceuticals ineffectively cross cellular membranes. Second, unlike kinase, enzymes and receptors, transcription factors do not simply bind to small-molecular ligand directly. Thus, it will be very challenging to screen out a direct ETV2 inhibitor, and extracellular signals that activate Etv2 should be better therapeutic targets than ETV2 itself. Given the extremely complex and dynamic microenvironment of GBM, it would be interesting to determine whether the reactivation of Etv2 in the primary GBM neural stem-like cells is also dependent on BMP, Notch and Wnt signaling.

Several studies have shown that some ECs (ranging from 20 to 90%) in GBM are malignant and directly derived from the transdifferentiation of GBM tumor cells. For example, the CD133+/CD144+ population of GBM tumor cells can differentiate into endothelial cells in endothelial culture medium.^8^ Similarly, another group found that CD31+ GBM tumor cells are capable of differentiating into endothelial cells that support tumor angiogenesis. These TDECs are genetically abnormal and refractory to inhibition of the VEGF-A and basic fibroblast growth factor (bFGF and FGF-2) pathways.^2^ Our studies offer a role for Etv2 as a possible underlying contributor to the observed transdifferentiation of GBM to vascular endothelial cells. The finding that the ETV2 level is correlated with GBM malignancy suggests that its expression, determined by mRNA or protein levels, can be used as a diagnostic and prognostic indicator. Further studies utilizing a larger population size are warranted to determine these applications of ETV2.

Although many transcription factors have been shown to be related to vascular development, none appear to be as centrally required as ETV2 during the early differentiation of endothelial cells.^12, 29, 32^ The ETS family transcription factor Etv2 (also known as Etsrp or ER71) is an evolutionarily conserved early mediator of vascular and hematopoietic development. We first identified the function of this gene in zebrafish studies and then discovered its homologous pivotal role in mammals.^12, 29^ Since then, studies from multiple laboratories have established Etv2 as a master regulator of normal vascular development in vivo and ES cell differentiation to ECs in vitro, as well as a mediator of the transdifferentiation of several somatic cell types to endothelial cells.^15–17^ Studies herein represent the first case of Etv2 involvement in pathogenesis. It is worth noting that initial observations of neural stem cell transdifferentiation were first obtained in zebrafish studies, underscoring the value of this model organism for the study of human diseases.

## Methods

### Human GBM specimens

Biopsies of human brain tumor and normal tissues were provided by the West China Hospital, Sichuan University. Histological examination was performed by at least two experienced neuropathologists. The ETV2 expression level was analyzed in a total of 222 brain tumor specimens, including glioblastoma (GBM grade III–IV, *n* = 81), astrocytoma (grade I–II, *n* = 86), meningioma (grade I–II, *n* = 48), and oligodendroglioma (grade I–II, *n* = 7) in tissue microarrays (HBra-Gli065PG, GL2082; US Biomax). The prognostic value of the Etv2 expression level was analyzed according to previously published microarray data and clinical follow-up information from 75 grade III–IV brain tumor samples acquired from M.D. Anderson Cancer Center (MDA) and UCSF. Data were re-analyzed by Kaplan–Meier analysis and the log-rank test.^19, 20^ The raw data for the selected genes were downloaded and analyzed in Prism (GraphPad Software) to generate the survival curve. Utilization of all human specimens was performed in accordance with the ethics commissions of the West China Hospital, Sichuan University, China.

### GBM cells

Primary GBM tumor cells GBM #109, GBM #723, GBM #527, and GBM #24 were derived from GBM patients from West China Hospital and prepared according to a previously published method.^20^ The GBM cell lines A172, U251, and U87 were obtained from the American Type Culture Collection (ATCC). For GBM neural stem cell enrichment, cells were cultured as spheroids; cells were seeded in 2% poly(2-hydroxyethyl methacrylate) (poly-HEMA, Sigma)-coated cell culture dishes. Tumor spheres were formed in serum-free DMEM/F12 medium supplemented with N2 supplement and 0.5 mg/ml BSA, 20 ng/ml EGF and bFGF. For passaging the spheroids, 100–150 μm spheres were dissociated in 0.05% trypsin-EDTA and seeded at a density of 10,000 cells/ml. The cell culture medium was changed every 3–4 days. After culturing for 3 passages under neural stem cell enrichment conditions, GBM tumor cells are infected with lentivirus with ETV2-YFP (LV-ETV2-YFP) or control LV-YFP and cultured either in basic or complete (supplemented with angiogenic growth factors) endothelial growth medium EGM (Lonza, Clonetics). For differentiation and differentiated tumor cell culturing, tumor cells were grown as monolayers with DMEM containing 10% (v/v) fetal bovine serum (FBS), 2 mM l-glutamine, 100 IU/ml penicillin, 100 μg/ml streptomycin, and 1% nonessential amino acid. All cells were cultured at 37 °C in a humidified atmosphere of 5% CO_2_. Cell culture medium and additives were obtained from Invitrogen.

### Transgenic zebrafish and heat shock

The lines used in this study were flia:GFP (zfin: y1Tg), kdrl: mCherry (zfin: zf527Tg), hs70i:Etv2 (zfin: zf434Tg), and GFAP: GFP (zfin: mi2001Tg). Etv2 in hs70i:Etv2 zebrafish was fused to mCherry, while the heat shock-induced Etv2-mCherry fluorescence was weak and transient (generally detected for 4 h). Thus, we denoted this line as hs70i:Etv2. Heat shock was performed at 38.5 °C by placing the zebrafish embryos in a 3-cm plastic dish with 2 mL of water, sealing the dish with parafilm, and floating it on the surface of a 38.5 °C water bath for 30 min. The dish was then placed at 28.5 °C until the desired time points for analysis. All the fish used were bred and maintained normally (temperature, 28 °C; pH 7.2–7.4; 14-h:10-h light:dark cycle).

### Immunostaining and in situ hybridization

Immunostaining was performed using 8-μm cryosections as previously described.^16^ Antibodies in this study were anti-ETV2 (N-term) (AP11311a, ABGENT), anti-ETV2 (C-term) (AB65825, Abcam), anti-ETV2(Internal)(LS-C61735, Isbio), anti-hCD31 (AB32457, Abcam), anti-CD31 (AB9498, AB28365, Abcam), anti-ki67 (AB156956, AB15580, Abcam), anti-CD34 (AB157304, Abcam), anti-VE-cad (AB7047, Abcam), anti-flk1 (#2478, CST), anti-nestin (ab6320, Abcam), anti-CD133 (AB19898, Abcam), anti-GFAP (AB7260, Abcam), anti-NeuN (SAB4300883, Sigma), and anti-Sox2 (AB97959, Abcam). Sections were mounted using Prolong Gold with DAPI (Life Technologies). In situ hybridization was performed as previously described.^16^

### hETV2 knockdown

Three gRNAs were designed based on the hETV2 sequence (ETS variant 2, ID2116, GeneBank, NCBI). Two functional 20-base nucleotides (CGTGGTCGCGCGCCCAGGC, GCAGCTGCATCCGTTGGAC) were cloned into the gRNA vector using a Gibson assembly kit (New England BioLabs, Ipswich, MA) according to the manufacturer’s protocol to construct ETV2-specific gRNA-expression vectors. The fragment containing the U6 promoter and ETV2-specific gRNA was PCR-amplified from the individual gRNA-expression vector and subsequently inserted into the hCas9 expression lentivirus vector to construct the ETV2-specific gRNA/hCas9 dual expression Lentivirus package vector. To demonstrate CRISPR/Cas9-mediated cleavage, we applied T7E1 (New England Biolabs) to digest imperfectly matched DNA after hybridization of the DNA mixture derived from two U87 GBM tumor cell clones (E5, E6). PCR products were amplified by ETV2-S-F:GAGGGCTAAGAAACTGGTAGTC; ETV2-S-R:ATTCATGCCCGGCTTTCT and subjected to the T7E1 assay according to the manufacturer’s protocol.

### ChIP-Seq

Homozygous heat shock hsp70l:etv2 fish were out-crossed with wild-type fish, exclusively producing embryos with ubiquitous heat-inducible C-terminal mCherry-tagged Etv2, and equal numbers of wild-type in-crosses were processed in parallel with controls. ChIP was performed with a Protein A ChIP kit (Millipore) according to the manufacturer’s instructions with some modifications prior to sonication (see also supplemental material and methods). Each ChIP sample was resuspended in 40 μl elution buffer (QIAGEN) after chloroform extraction, and 2 μl of each was tested by qPCR with SYBR green (Roche) using the primers listed in Table S4. Enrichment was determined by the ΔΔCt method and standardized with rhodopsin primers published previously.^33^

### RNA-Seq

For Etv2 overexpression, 100 embryos of either wild-type controls or hsp70l-etv2-mCherry embryos were heat shocked for 30 min and re-incubated for 6 h at 28 °C before harvesting for library preparation. Total RNA for all samples was harvested with TRIzol (Life Technologies) and further purified with an Rneasy kit (QIAGEN). RNA-Seq libraries were constructed with the TRU-Seq preparation kit according to the manufacturer’s instructions (Illumina).

### Bioinformatics

A detailed description of the data processing is provided in Supplemental Bioinformatics Methods.

### Statistical analysis

An unpaired, one-tailed Student’s *t* test was applied with GraphPad Prism 5 software to compute the difference between two independent groups. *P* value, *<0.05, **<0.01, ***<0.001. Unless noted otherwise, data are reported as the mean ± s.e.m.

## Electronic supplementary material

Supplementary material

Supplementary Tables

## References

[1] Stupp R (2005). Radiotherapy plus concomitant and adjuvant temozolomide for glioblastoma. N. Engl. J. Med..

[2] Wang R (2010). Glioblastoma stem-like cells give rise to tumour endothelium. Nature.

[3] Han T, Kim JK (2014). Driving glioblastoma growth by alternative polyadenylation. Cell Res..

[4] Kreisl TN (2009). Phase II trial of single-agent bevacizumab followed by bevacizumab plus irinotecan at tumor progression in recurrent glioblastoma. J. Clin. Oncol..

[5] Hormigo A, Ding BS, Rafii S (2011). A target for antiangiogenic therapy: vascular endothelium derived from glioblastoma. Proc. Natl Acad. Sci. USA.

[6] Jain RK (2005). Normalization of tumor vasculature: an emerging concept in antiangiogenic therapy. Science.

[7] Jain RK (2007). Angiogenesis in brain tumours. Nat. Rev. Neurosci..

[8] Ricci-Vitiani L (2010). Tumour vascularization via endothelial differentiation of glioblastoma stem-like cells. Nature.

[9] Soda Y (2011). Transdifferentiation of glioblastoma cells into vascular endothelial cells. Proc. Natl Acad. Sci. USA.

[10] Ben-Porath I (2008). An embryonic stem cell-like gene expression signature in poorly differentiated aggressive human tumors. Nat. Genet..

[11] Takahashi K, Ichisaka T, Yamanaka S (2006). Identification of genes involved in tumor-like properties of embryonic stem cells. Methods Mol. Biol..

[12] Sumanas S, Lin S (2006). Ets1-related protein is a key regulator of vasculogenesis in zebrafish. PLoS Biol..

[13] Dejana E, Taddei A, Randi AM (2007). Foxs and Ets in the transcriptional regulation of endothelial cell differentiation and angiogenesis. Biochim. Biophys. Acta.

[14] Murakami Y (2006). Ets-1-dependent expression of vascular endothelial growth factor receptors is activated by latency-associated nuclear antigen of Kaposi’s sarcoma-associated herpesvirus through interaction with Daxx. J. Biol. Chem..

[15] Morita R (2015). ETS transcription factor ETV2 directly converts human fibroblasts into functional endothelial cells. Proc. Natl Acad. Sci. USA.

[16] Veldman MB (2013). Transdifferentiation of fast skeletal muscle into functional endothelium in vivo by transcription factor Etv2. PLoS Biol..

[17] Ginsberg M (2012). Efficient direct reprogramming of mature amniotic cells into endothelial cells by ETS factors and TGFbeta suppression. Cell.

[18] Lam CS, Marz M, Strahle U (2009). gfap and nestin reporter lines reveal characteristics of neural progenitors in the adult zebrafish brain. Dev. Dyn..

[19] Phillips HS (2006). Molecular subclasses of high-grade glioma predict prognosis, delineate a pattern of disease progression, and resemble stages in neurogenesis. Cancer Cell.

[20] Lee J (2006). Tumor stem cells derived from glioblastomas cultured in bFGF and EGF more closely mirror the phenotype and genotype of primary tumors than do serum-cultured cell lines. Cancer Cell.

[21] Baumann, B. C., Dorsey, J. F., Benci, J. L., Joh, D. Y., & Kao, G. D. Stereotactic intracranial implantation and in vivo bioluminescent imaging of tumor xenografts in a mouse model system of glioblastoma multiforme. *J. Vis. Exp.*10.3791/4089 (2012). 10.3791/4089 PMC3490274 23051742

[22] David MD, Canti C, Herreros J (2010). Wnt-3a and Wnt-3 differently stimulate proliferation and neurogenesis of spinal neural precursors and promote neurite outgrowth by canonical signaling. J. Neurosci. Res..

[23] Lopez-Juarez A (2013). Gsx2 controls region-specific activation of neural stem cells and injury-induced neurogenesis in the adult subventricular zone. Genes Dev..

[24] Gunsilius E (2000). Evidence from a leukaemia model for maintenance of vascular endothelium by bone-marrow-derived endothelial cells. Lancet.

[25] Pezzolo A (2007). Tumor origin of endothelial cells in human neuroblastoma. J. Clin. Oncol..

[26] Rigolin GM (2006). Neoplastic circulating endothelial cells in multiple myeloma with 13q14 deletion. Blood.

[27] Cao Z (2014). Angiocrine factors deployed by tumor vascular niche induce B cell lymphoma invasiveness and chemoresistance. Cancer Cell.

[28] Butler JM, Kobayashi H, Rafii S (2010). Instructive role of the vascular niche in promoting tumour growth and tissue repair by angiocrine factors. Nat. Rev. Cancer.

[29] Lee D (2008). ER71 acts downstream of BMP, Notch, and Wnt signaling in blood and vessel progenitor specification. Cell Stem Cell.

[30] Kaur N (2013). Wnt3a mediated activation of Wnt/beta-catenin signaling promotes tumor progression in glioblastoma. Mol. Cell Neurosci..

[31] Kanamori M (2007). Contribution of Notch signaling activation to human glioblastoma multiforme. J. Neurosurg..

[32] Koyano-Nakagawa N (2012). Etv2 is expressed in the yolk sac hematopoietic and endothelial progenitors and regulates Lmo2 gene expression. Stem Cells.

[33] Wardle FC (2006). Zebrafish promoter microarrays identify actively transcribed embryonic genes. Genome Biol..

