# Supplementary material for: ETV2 mediates endothelial transdifferentiation of glioblastoma
Source: Signal Transduct Target Ther. 2018 Feb 9;3:4. doi: 10.1038/s41392-018-0007-8 (PMC5837107; doi:10.1038/s41392-018-0007-8)
Supplement: Supplementary file 1 — Supplementary material [file 41392_2018_7_MOESM1_ESM.pdf]

Supplementary material:

ETV2 mediates endothelial transdifferentiation of glioblastoma

Chengjian Zhao[1], Gustavo A. Gomez[1], Yuwei Zhao[3], Yu Yang[2], Cao Dan[2], Jing Lu[1], Hanshuo Yang[2,*], and Shuo Lin[1,*]

1. Department of Molecular, Cell and Developmental Biology, University of California - Los Angeles, Los Angeles, CA 90095, USA

2. State Key Laboratory of Biotherapy and Cancer Center, West China Hospital, Sichuan University, and Collaborative Innovation Center for Biotherapy, Sichuan, P. R. China

3. Blood Research Laboratory, Chengdu blood Center, Sichuan, P. R. China

* Correspondence E-mail: yhansh@126.com and shuolin@ucla.edu

Supplementary materials including 9 Figures and Bioinformatics Methods

Fig S1

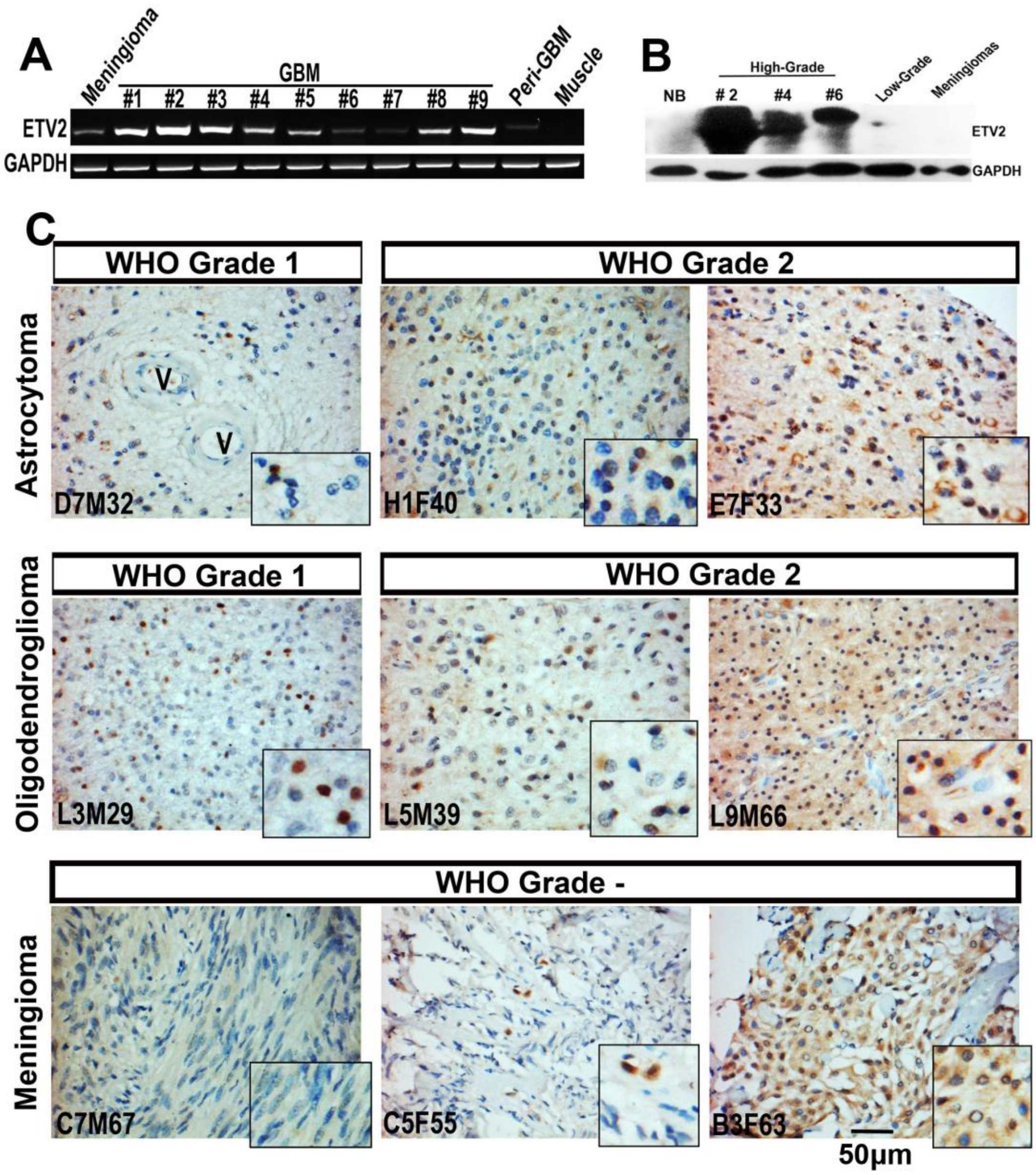

Fig S1. (A)RT-PCR shows the transcription of ETV2 in high-grade brain tumors and normal tissue. (#1-#9: grade IV GBM from West china hospital, Peri-GBM: peripheral normal tissue of GBM#9, muscle: Skeletal muscle). (B) Western-blot shows the expression of ETV2 in high-grade brain tumors and normal tissue. NB: normal brain tissue. (C) IHC staining for ETV2 of human brain tumors (Astrocytoma gradeI-II, n=86; Meningioma gradeI-II, n=48; Oligdendroglioma gradeI-II, n=7); representative areas were magnified; V: vessels.

Fig S2

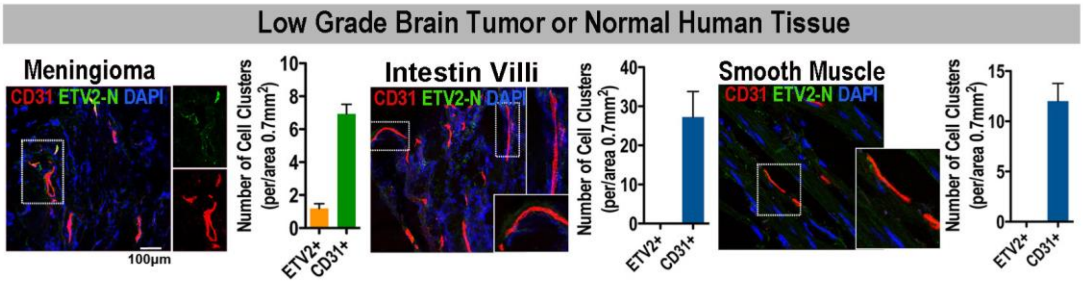

Fig S2. Multicolor IF staining for ETV2 and CD31 using Meningioma, intestin Villi and smooth muscle tissues from west china hospital. The number of ETV2+ clusters in each viewing area were quantified right. areas in dotted boxes were magnified.

Fig S3

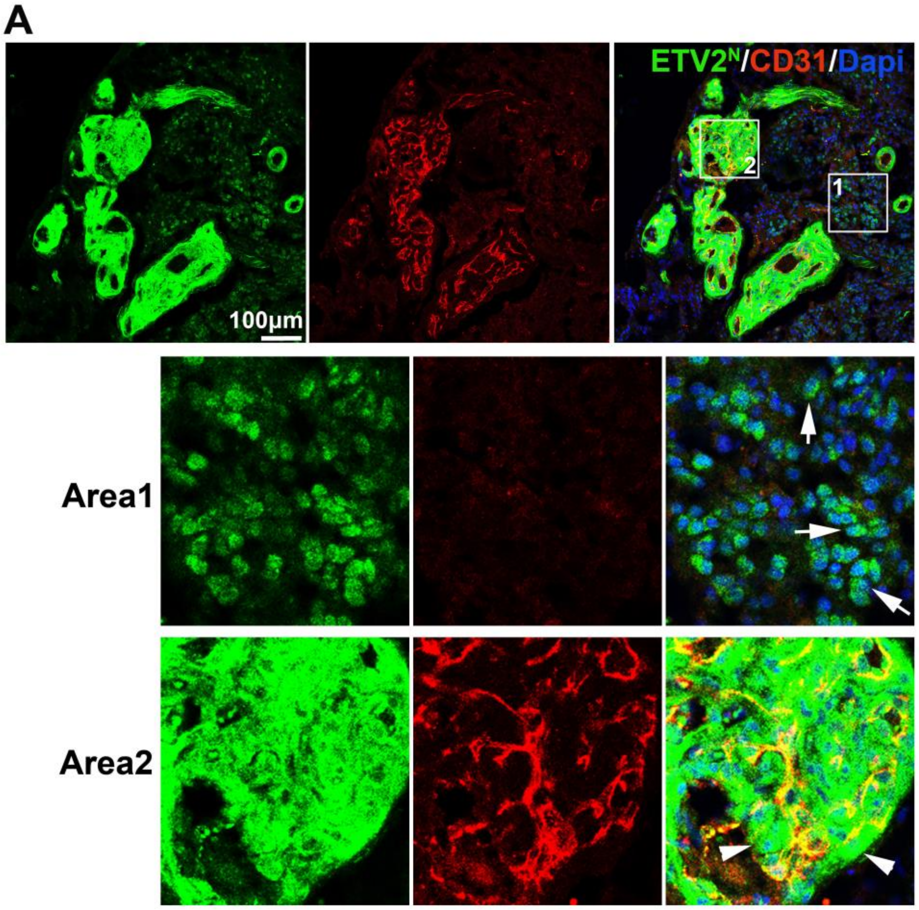

Fig S3. Multicolor IF staining for ETV2-N and CD31 using freshly collected grade-IV GBM tissue (#109); areas in dotted boxes were magnified below. Arrows indicate ETV2 expression in nucleus of GBM tumor cells, arrowheads indicate ETV2 expression in the cytoplasm of GBM tumor cells.

Fig S4

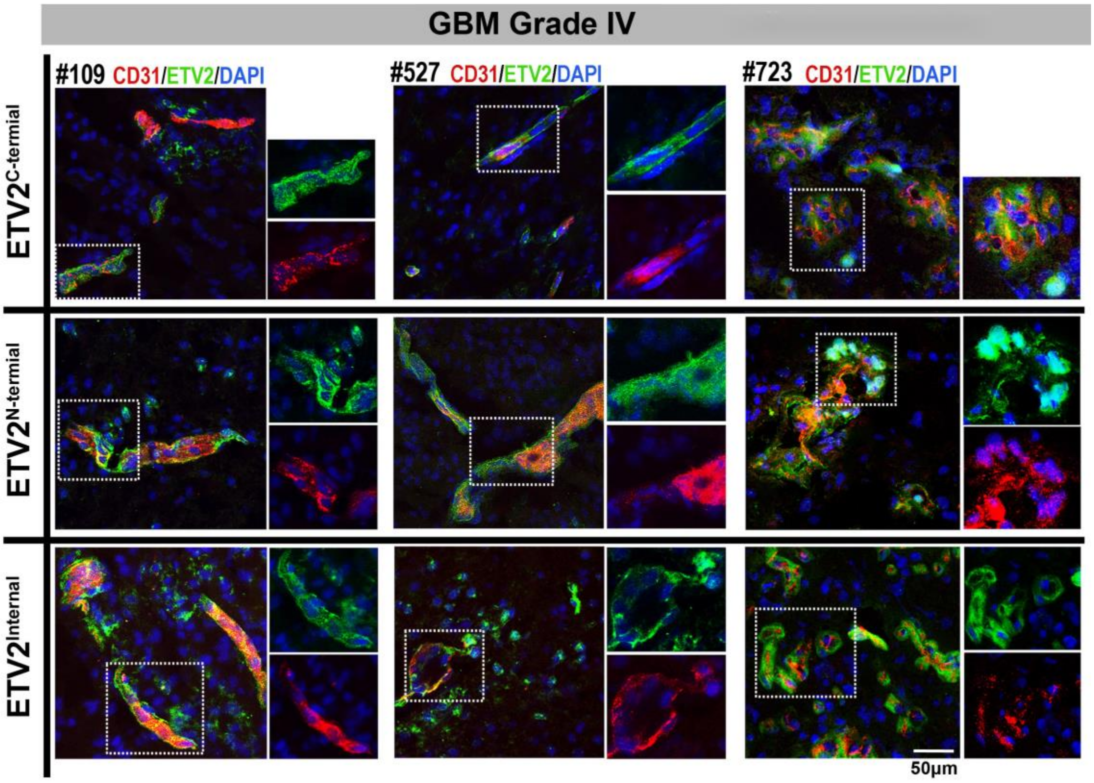

Fig S4. Multicolor IF staining for ETV2 (ETV2-C-terminal; ETV2-N-terminal; ETV2-internal) and CD31 using freshly collected grade-IV GBM tissue (#109, #527, #723); areas in dotted boxes were magnified right.

Fig S5

A

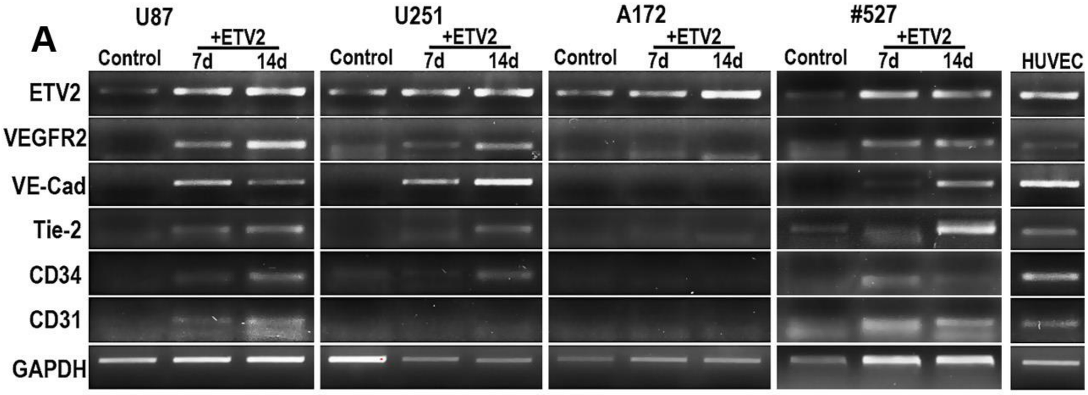

B

EC-related	Cells with ETV2-OE							
	U87	U251	A172	#527	#723	#109	#24	293-HEK
FLK/Vegfr2	+	+	-	+	+	+	+	-
VE-Cad	+	+	-	+	+	+	+	-
Tie-2	+	-	-	+	+	+	+	-
CD34	+	-	-	+	+	+	+	-
CD31	+	-	-	+	+	+	+	-

C

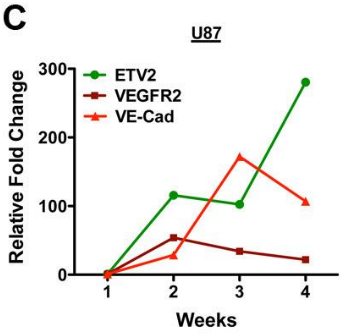

Fig S5. (A-C)RT-PCR and q-PCR show the induction of endothelial genes in GBM tumor cells (U87, U251, A172 and patient's GBM#527) after ETV2 overexpression. GBM tumor cells (#109, #723, #527, A172, U251, U87) were propagated as neural-spheres under conditions optimal for neural stem cells. LV-ETV2 infection was used to overexpress ETV2 in these cells, cells were then cultured in endothelial growth medium (complete EGM) before evaluation. 1 week to 4 weeks after ETV2 overexpression, RT-PCR (A, B) and q-PCR (C) were performed to evaluate the expression of endothelial genes in these GBM cells.

Fig S6

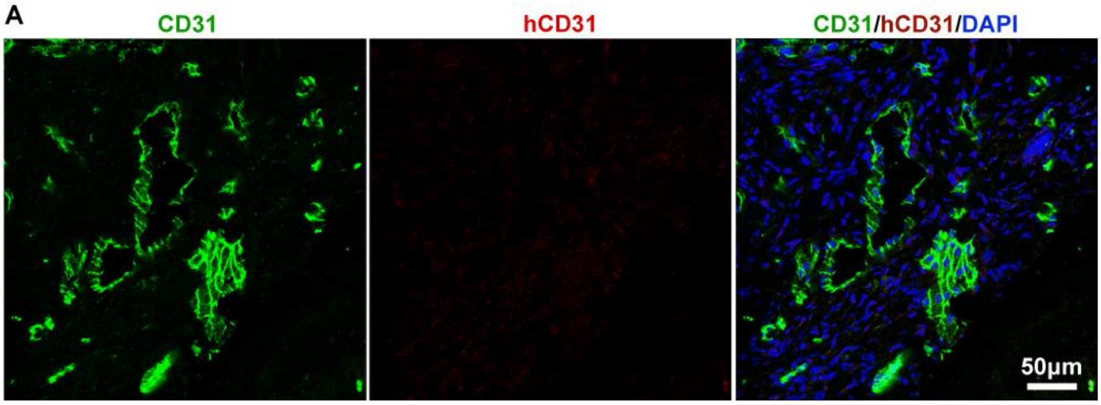

Fig S6. (A) Multicolor IF staining of a CT26 mouse subcutaneous tumor indicated that the hCD31 antibody (ab32457) didn’t recognize mouse endothelial cells (CD31+, ab9498).

Fig S7

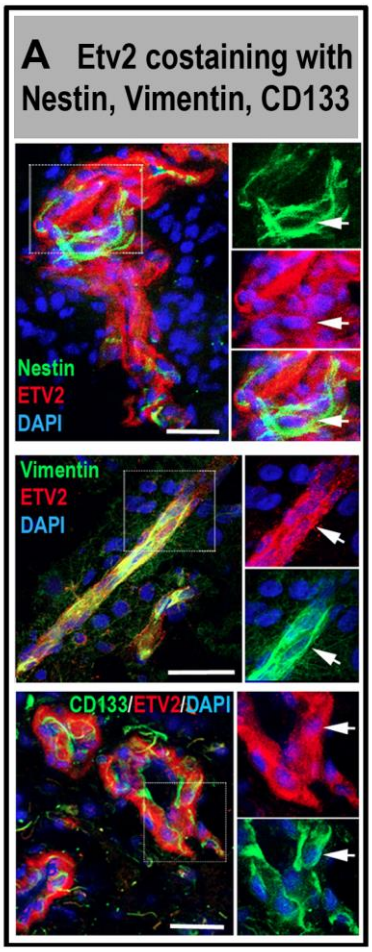

Fig S7. (A) Multicolor IF staining shows the ETV2 positive GBM tumor cells (Patient #109, arrows) were co-expressing with neural stem cell marker genes (Nestin, Vimentin and CD133). Scale bar: 50 µm.

Fig S8

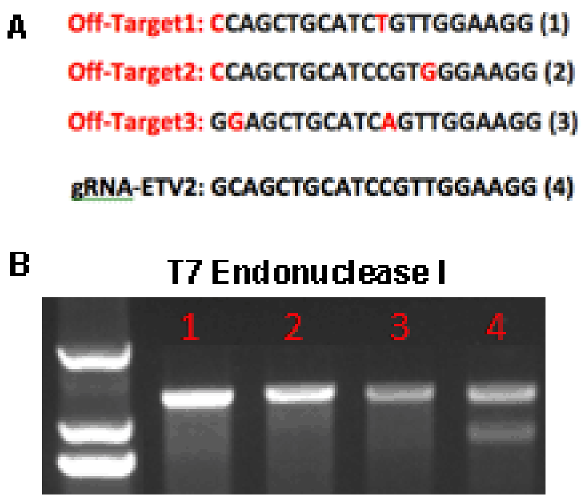

Fig S8. (A) 3 potential off-target sites of gRNA-ETV2 have been identified. (B) T7 EI assay indicated that ETV2-gRNA didn’t induce mutations at potential off-target sites.

Fig S9

A **Neural Genes Reduced by ETV2 Overexpression Below 2-fold That Contain ChIP-seq Peaks**

Gene Symbol	Description	Expression Area
acsl4a	acyl-CoA synthetase long-chain family member 4a	cns and ysl
her5	hairy-related 5	midbrain hindbrain boundary
her5	hairy-related 5	midbrain hindbrain boundary
sema3gb	semaphorin 3gb	cns
ptf1a	pancreas specific transcription factor, 1a	cns at stages examinec
lbx1a	ladybird homeobox 1a	cns
scrt1a	scratch homolog 1, zinc finger protein a (Drosophila)	cns
gsx1	GS homeobox 1	cns
wnt3	wingless-type MMTV integration site family, member 3	neural tube
speg	SPEG complex locus	cns
sox19b	SRY-box containing gene 19b	spinal chord, cns
wnt10b	wingless-type MMTV integration site family, member 10b	cns
dkk1b	dickkopf 1b	cns
pdzrn4	PDZ domain containing ring finger 4	cns, spinal cord
atoh1a	atonal homolog 1a	cns
lhx5	LIM homeobox 5	cns
lama5	laminin, alpha 5	cns, and other
etv5a	ets variant 5a	spinal chord and pronephros
prdm13	PR domain containing 13	spinal chord and neurc
ascl1b	achaete-scute complex-like 1b (Drosophila)	cns

Fig S9. (A) Ectopic Etv2 overexpression repression 798 genes relative to wild type controls. Of the 798 genes, 111 contain ChIP-Seq peaks, and although the expression patterns of only 57 of these have been documented at the developmental stages studied here (between 15 and 21 hpf), 24 are expressed in somites, 20 are expressed in the nervous system.

Supplementary Chip-seq method

ChIP-seq

Binding of the mCherry antibody (rabbit anti-DS Red polyclonal, Clontech) to Etv2-mCherry was tested by whole mount fluorescent immunostaining before performing ChIP-seq experiments on progeny from a *hsp70l:etv2-mCherry* heterozygote line outcrossed to a wild type line, producing half a clutch with the inducible *etv2-mCherry* transgene and half without. The primary antibody was tested at 1:500 followed by 1:1000 of the Alexa Fluor 488 anti-rabbit secondary IgG (A11008 Life Technologies).

For ChIP, homozygous heat shock *hsp70l:etv2-mCherry* fish were out-crossed with wild-type fish, exclusively producing embryos with ubiquitous heat inducible C-terminal mCherry tagged Etv2, and equal numbers of wild type in-crosses were processed in parallel as controls. Transgenic expression was stimulated by floating embryos in Petri dishes at 38.5° C for 45 minutes, followed by re-incubation for 1 hour at 28.5° C before harvesting. ChIP was performed with the Protein A ChIP kit (Millipore) according to the manufacturer's instructions with the following modifications prior to sonication. Cells from 200 embryos per ChIP were first de-yolked with Ringer's solution containing 0.5M EDTA with 2 cycles of centrifugation at 1,000Xg between changes of Ringer's buffer before fixation in 1% formaldehyde for 10 minutes. Samples were quenched with 0.125M Glycine, followed by 3 washes in PBS with protease inhibitors (Roche) and stored at -80°C. Cells were lysed in cell lysis buffer (10mM Hepes pH7.9, 0.5% NP40, 1.5mM MgCl2, 10mM KCl, 0.5mM DTT), followed by a high salt wash (50mM Hepes pH7.9, 0.1% SDS, 1% Triton X-100, 0.1% deoxycholate, 1mM EDTA, 500mM NaCl), and a final lysis in nuclear lysis buffer (1% SDS, 50mM Tris-HCl pH8, 20mM EDTA). Each lysis step was performed by passing samples through a 200 µl pipette tip and a 10 minute incubation on ice followed by a 10 minute centrifugation step (2,000Xg) at 4°C. Thereafter, lysates were sonicated with a standard Bioruptor (Diagenode) at a high frequency setting for 2 cycles, each cycle lasting 10 minutes with each minute divided into 30 seconds of sonication followed by 30 seconds of rest. Ice was changed between cycles to prevent overheating and denaturation of samples, producing a final sheared protein bound-DNA range of 200-500bp. The anti-DS Red polyclonal antibody (Clontech) was used at 5 ug per ChIP. A

total of 1200 embryos were used for each ChIP-seq library. 2ng of each sample was used to generate each library with the Ovation Ultralow IL Multiplex System (Nugen) according to manufacturer's instructions. The final libraries were gel extracted with mini-elute columns (QIAGEN) at a range of 300-550 bp. Libraries were processed on an Illumina Hi-seq 2000 sequencer to generate single end 50bp reads.

Each ChIP sample was resuspended in 40µl elution buffer (QIAGEN) after chloroform extraction, and 2µl of each was tested by qPCR with SYBR green (Roche) using the primers listed in table $$1. Enrichment was determined by the ΔΔ Ct method and standardized with *rhodopsin* primers that were published previously[1].

Reference:

1. Wardle, F.C., *et al.* Zebrafish promoter microarrays identify actively transcribed embryonic genes. *Genome biology* **7**, R71 (2006).

Supplementary Methods: Bioinformatics

RNA-seq

The Illumina HiSeq 2000 platform yielded approximately 150 ~ 215 million single end short reads (50bp) per sample (Tables SM1 and SM2). Bowtie (version 0.12.8) (Langmead et al., 2009) was used to align the reads to the zebrafish genome Zv9 (danRer7) with up to 2 mismatches allowed. Each sample yielded mappable reads in excess of 80% (Tables SM1 and SM2), however, only uniquely mapped reads were used in subsequent gene differential expression analysis. Read counts per gene were summarized by the R package, GenomicRanges coupled with the UCSC danRer7 gene annotation table ensGene. A standard pair-wise comparison of two samples was performed by DESeq (Anders and Huber, 2010) with default parameters.

Table SM1

Sample	Total raw reads	Total uniquely mapped reads	Total multiple mapped reads	Total reads not mapped
hsp70l-Etv2-mCherry wild type	214,241,173	138,342,349 (64.57%)	19,552,429 (9.13%)	56,346,395 (26.30%)
***hsp70l-Etv2-mCherry* OE**	217,217,421	140,770,907 (64.81%)	17,955,469 (8.27%)	58,491,045 (26.93%)

ChIP-seq

ChIP-seq DNA samples from wild type and *hsp701:etv2-mCherry* heterozygous zebrafish were sequenced separately in 2 lanes on the Illumina Hiseq 2000 platform. Overall, 82,311,932 short reads (50bp) were obtained from the wild type control sample and 55,560,402 short reads were obtained from *etv2-mCherry*. The R package, FastQC, was utilized to perform a sequencing quality check. The first 4 bases corresponding to the inline barcodes were trimmed from the raw reads before mapping. Bowtie (--sam --best –n 2 –m 1) (Langmead et al., 2009) was used to map the trimmed raw reads to the zebrafish genome Zv9. Approximately 17 million and 13 million uniquely mapped reads for wild type and etv2-mCherry samples were identified respectively (Table SM2). MACS (version 1.4) (Zhang et al., 2008) was then used to identify the corresponding read enriched regions on the genome with the following parameters: -t etv2_uniquly_mapped_reads -c control_uniquely_mapped_reads -g 1.5e9 -s 46 -w --single-profile --verbose 1. 10,029 peak regions were obtained with p-values < 1.00e-5.

Table SM2

Samples	Total raw reads	Total uniquely mapped reads	Total multiple mapped reads	Total reads not mapped
Etv2-mCherry **ChIP-seq**	55,560,402	13,407,569 (24.13%)	7,694,800 (13.85%)	34,458,033 (62.02%)
wild type **ChIP-seq**	82,311,932	16,905,309 (20.54%)	11,068,679 (13.45%)	54,337,944 (66.01%)

The R Package ChIPpeakAnno (Zhu et al., 2010) and biomaRt were used to annotate the peaks regions. Ensembl (version 69) annotated zebrafish Transcription Start Sites(TSSs) were utilized in ChipPeakAnno to perform the annotation step. 6,950 Ensembl zebrafish TSSs were found to be neighboring the aforementioned 10,029 peak regions (Supplementary Table 5). Among these, 9,687 peak regions were within 10Kb of the nearest TSS, and 6,856/6,950 TSSs were within 10 KB of a peak region. A +/- 10Kb flanking distance distribution to these TSSs indicated that most Etv2-mCherry binding sites were within several hundred base pairs of the TSSs, and a bimodal distribution flanking these TSSs can also be observed (below).

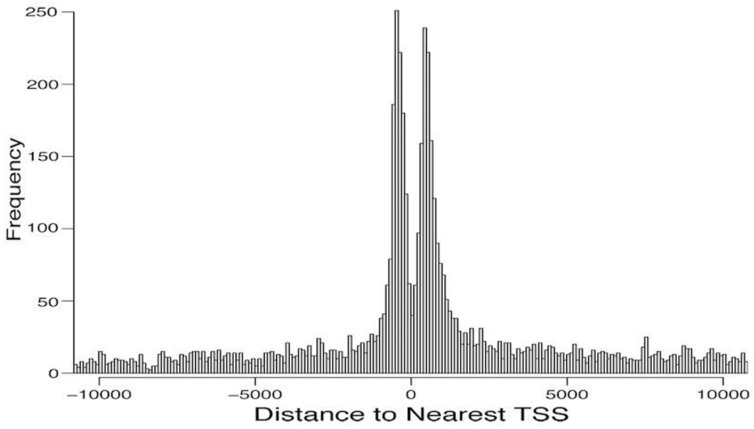

Genomic sequences +/- 100bp from the summits of peaks enriched by MACS were extracted and submitted to DREME (Bailey, 2011) to investigate possible motifs in our Etv2-mCherry ChIP-seq data.

References:

Anders, S., and Huber, W. (2010). Differential expression analysis for sequence count data. Genome Biol *11*, R106.
Bailey, T.L. (2011). DREME: motif discovery in transcription factor ChIP-seq data. Bioinformatics *27*, 1653-1659.

Langmead, B., Trapnell, C., Pop, M., and Salzberg, S.L. (2009). Ultrafast and memory-efficient alignment of short DNA sequences to the human genome. Genome Biol *10*, R25.
Zhang, Y., Liu, T., Meyer, C.A., Eeckhoute, J., Johnson, D.S., Bernstein, B.E., Nusbaum, C., Myers, R.M., Brown, M., Li, W., *et al.* (2008). Model-based analysis of ChIP-Seq (MACS). Genome Biol *9*, R137.
Zhu, L.J., Gazin, C., Lawson, N.D., Pagès, H., Lin, S.M., Lapointe, D.S., and Green, M.R. (2010). ChIPpeakAnno: a Bioconductor package to annotate ChIP-seq and ChIP-chip data. In BMC Bioinformatics, pp. 237.
